# Advanced Morphological and Material Engineering for High‐Performance Interfacial Iontronic Pressure Sensors

**DOI:** 10.1002/advs.202413141

**Published:** 2025-01-22

**Authors:** Fengling Zhuo, Zhi Ding, Xi Yang, Fengjian Chu, Yulu Liu, Zhuoqing Gao, Hao Jin, Shurong Dong, Xiaozhi Wang, Jikui Luo

**Affiliations:** ^1^ College of Information Science and Electronic Engineering Zhejiang University Hangzhou 310027 China; ^2^ International Joint Innovation Center Zhejiang University Haining 314400 China; ^3^ Research Institute of Medical and Biological Engineering Ningbo University Ningbo 315211 China

**Keywords:** flexible EDL pressure sensor, morphological engineering, sensing performance, fabrication techniques, advanced applications

## Abstract

High‐performance flexible pressure sensors are crucial for applications such as wearable electronics, interactive systems, and healthcare technologies. Among these, iontronic pressure sensors have garnered particular attention due to their superior sensitivity, enabled by the giant capacitance variation of the electric double layer (EDL) at the ionic‐electronic interface under deformation. Key advancements, such as incorporating microstructures into ionic layers and employing diverse materials, have significantly improved sensor properties like sensitivity, accuracy, stability, and response time. This review highlights advancements in flexible EDL pressure sensors, focusing on structural designs and material engineering. These strategies are tailored to optimize key metrics such as sensitivity, detection limit, linearity, stability, response speed, hysteresis, transparency, wearability, selectivity, and multifunctionality. Key fabrication techniques, including micropatterning and externally assisted methods, are reviewed, along with strategies for sensor comparison and guidelines for selecting appropriate sensors. Emerging applications in healthcare, environmental and aerodynamic sensing, human–machine interaction, robotics, and machine learning‐assisted intelligent sensing are explored. Finally, this review discusses the challenges and future directions for advancing EDL‐based pressure sensors.

## Graphical abstract

This review provides a comprehensive framework for flexible iontronic pressure sensors, focusing on innovative structural and material engineering to achieve superior sensitivity, stability, and multifunctionality. It explores fabrication techniques, application‐driven sensor selection, and transformative use cases in healthcare, robotics, and intelligent sensing. The work addresses key challenges and offers forward‐looking insights into next‐generation EDL sensor technologies.

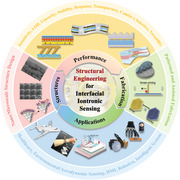

## 1 Introduction

The rapid advancement of flexible electronics and wearable technologies has raised a significant demand for high‐performance flexible pressure and tactile sensors. These sensors, converting physical or chemical stimuli into electrical signals, are indispensable components in applications such as wearable and implantable medical devices,^[^ ^1^ ^]^ human–machine interfaces,^[^ ^2^ ^]^ and artificial intelligence systems.^[^ ^3^ ^]^ Based on the mechanical‐to‐electrical signal conversion mechanisms, flexible pressure sensors can be broadly classified into resistive,^[^ ^4^ ^]^ capacitive,^[^ ^5^ ^]^ piezoelectric,^[^ ^6^ ^]^ and triboelectric types.^[^ ^7^ ^]^ Among these, capacitive sensors are particularly attractive due to their simple structure, energy efficiency, fast response, and stability.^[^ ^8^ ^]^ However, conventional capacitive sensors face inherent limitations such as low sensitivity and narrow pressure‐response range.^[^ ^9^ ^]^ To address these challenges, the architectural engineering of interfacial structures within dielectric and composite elastomers has been explored.^[^ ^10^ ^]^ Introducing microstructures into dielectric elastomers can enhance the sensors’ sensitivity by increasing mechanical compliance and the effective dielectric constant. Additionally, creating voids within the material facilitates elastic deformation, thereby improving response speed.^[^ ^10^, ^11^ ^]^ Nevertheless, dielectric elastomers are largely incompressible and are typically limited to a thickness of ≈10 µm. As a result, the capacitance change is generally small (<100 pF),^[^ ^11b^ ^]^ thus constraining sensitivity to approximately 1 kPa^−1^.

To overcome the limitations of conventional capacitive sensors, interfacial iontronic sensing, a novel mechanism, was introduced by Pan's group in 2011.^[^ ^12^ ^]^ In contrast to conventional capacitive sensors, interfacial iontronic sensors utilize an ionic film to replace the traditional dielectric layer. When this ionic film interfaces with a conductive electrode, positive and negative charges accumulate at the electrode/ionic film interface, forming an electric double layer (EDL) with a thickness of ≈ 1 nm. This configuration leads to extraordinarily high capacitance due to the tiny charge separation distance at the interface, providing two key advantages. First, the significantly shorter charge separation distance in EDL sensors, compared to the conventional dielectric thickness of 10–100 µm, enhances unit area capacitance (UAC) by several orders of magnitude under applied pressures, resulting in substantially improved sensitivity.^[^ ^13^ ^]^ Second, the fixed charge separation within the EDL ensures that capacitance depends primarily on the contact area between the dielectric and the electrode, rather than the separation distance. This unique characteristic enables substantial sensitivity improvements through microstructural modifications.^[^ ^14^ ^]^ Moreover, this configuration minimizes parasitic influences along the transmission line and suppresses electromagnetic noises from the ambient environment, greatly enhancing the signal‐to‐noise ratio and overall sensor performance.^[^ ^12^, ^15^ ^]^ Moreover, EDL sensors exhibit high spatial resolution and effectively respond to both static and dynamic stimuli.^[^ ^13a^ ^]^

Recent research has focused on advancing EDL flexible pressure sensors by improving critical performance metrics, such as sensitivity, detection limit, linear range, and stability, to meet the demands of practical applications.^[^ ^13a^ ^]^ Various sensing materials with tailored microstructures have been developed to improve contact efficiency and signal conduction within the sensing layer and electrode. These structural features are categorized into nano‐ and microscale designs based on their dimensional properties. At the nano‐scale, functional mechanisms such as the ion pump effect^[^ ^16^ ^]^ and pseudocapacitive materials^[^ ^17^ ^]^ optimize material properties, while structural designs like graded stiffness^[^ ^18^ ^]^ and nanorod incorporation^[^ ^19^ ^]^ improve mechanical adaptability. At the micro‐scale, microroughness,^[^ ^20^ ^]^ internal porous structures,^[^ ^21^ ^]^ and multiscale hierarchical architectures^[^ ^22^ ^]^ improve stress management and boost sensor functionality. These microstructures are fabricated through patterned techniques (e.g., lithography and printing) and assisted methods (e.g., mechanical force‐assisted, heat‐induced, electric field‐assisted, gas bubble‐assisted, and template‐assisted).

**Figure 1:**
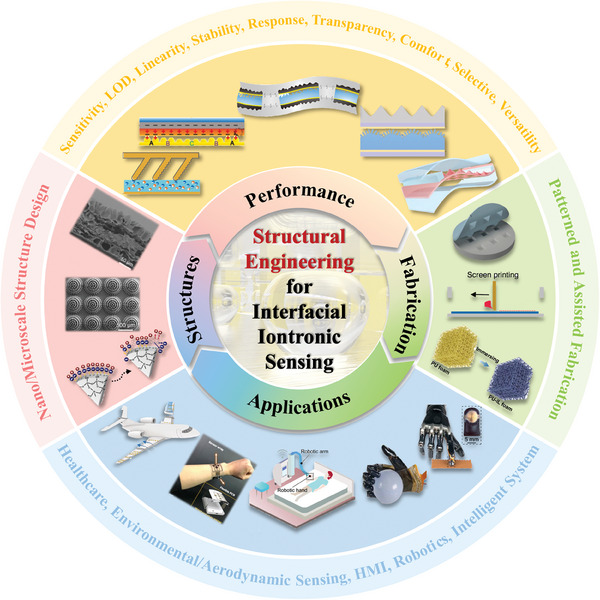
Overview of structure, properties, fabrication, and applications of EDL pressure sensor. Nano‐ and microscale structured sensing materials. Reproduced with permission.^[^ ^24^ ^]^ Copyright 2019, Springer Nature, Reproduced with permission.^[^ ^25^ ^]^ Copyright 2022, American Chemical Society, Reproduced with permission.^[^ ^26^ ^]^ Copyright 2018, WILEY‐VCH. Sensing performances include sensitivity, detection limit, linearity, stability, rapid response, transparency, wearability, and selectivity. Reproduced with permission.^[^ ^27^ ^]^ Copyright 2022, Yongsong Luo et al., Reproduced with permission.^[^ ^20b^ ^]^ Copyright 2023, Springer Nature, Reproduced with permission.^[^ ^28^ ^]^ Copyright 2023, The American Association for the Advancement of Science, Reproduced with permission.^[^ ^20c^ ^]^ Copyright 2023, Springer Nature, Reproduced with permission.^[^ ^29^ ^]^ Copyright 2021, The American Association for the Advancement of Science, and multifunctionality. Fabrication techniques include patterning and assisted methods. Reproduced with permission.^[^ ^16^ ^]^ Copyright 2023, Wiley‐VCH, Reproduced with permission.^[^ ^30^ ^]^ Copyright 2023, Springer Nature, Reproduced with permission.^[^ ^14^ ^]^ Copyright 2021, Springer Nature. Applications include health monitoring, environmental and aerodynamic sensing, human–machine interaction, robotics, and machine learning‐assisted intelligent sensing. Reproduced with permission.^[^ ^31^ ^]^ Copyright 2024, Springer Nature, Reproduced with permission.^[^ ^24^ ^]^ Copyright 2019, Springer Nature, Reproduced with permission.^[^ ^29^ ^]^ Copyright 2021, The American Association for the Advancement of Science, Reproduced with permission.^[^ ^28^ ^]^ Copyright 2023, The American Association for the Advancement of Science, Reproduced with permission.^[^ ^20c^ ^]^ Copyright 2023, Springer Nature.

Several review papers have summarized advancements in iontronic flexible sensors,^[^ ^13^, ^23^ ^]^ covering diverse mechanisms such as interfacial capacitive, piezoresistive, piezoelectric, triboelectric, mechano‐electroluminescent/electrochromic sensing, as well as emerging materials, structural designs, and applications. Among these, interfacial iontronic pressure sensors (EDL pressure sensors), which leverage the EDL effect, rely on mechanical deformation to modulate the interfacial contact area, enabling highly sensitive pressure detection. While some existing reviews have discussed structural designs for EDL pressure sensors, they often lack systematic frameworks for analyzing morphological engineering strategies. Key aspects, including the influence of multiscale structural designs (e.g., nanoscale architectures, microstructural configurations, or hierarchical morphologies) on critical performance metrics like sensitivity, detection limits, stability, and dynamic range, remain insufficiently explored. To address these gaps, this review systematically examines the role of morphological and material engineering in optimizing EDL‐based pressure sensors, providing a detailed framework that connects structural design with performance trade‐offs and multifunctionality. This review is structured as follows. Section 2 introduces the transduction mechanisms and material selection for EDL pressure sensors. Structural engineering of sensing layers is discussed in Section 3, followed by performance enhancements in Section 4. Section 5 reviews fabrication strategies for microstructures, while Section 6 provides guidelines for selecting appropriate sensors. Applications in wearable healthcare, environmental sensing, human–machine interaction, robotics, and ML‐enabled intelligent sensing platforms are presented in Section 7. Finally, conclusions and future prospects are outlined in Section 8. **Figure** **1** illustrates the overall structure and relationships between the sections.

## 2 Mechanisms and Materials for Iontronic Pressure Sensing

### 2.1 Electrical Double Layer Dynamics

**Figure 2:**
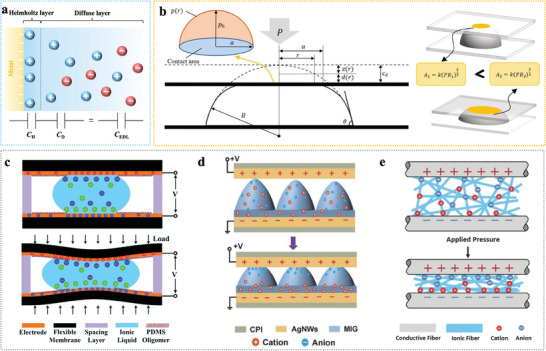
EDL model and typical interfacial iontronic sensing structures. a) Gouy‐Chapman‐Stern model.^[^ ^60^ ^]^ b) Deformation model of an elastic spherical cap structure and a rigid flat, analyzing the contact area between flats and spherical cap structures with different radii under the same pressures. Reproduced with permission.^[^ ^34^ ^]^ Copyright 2022, IOP Publishing. c) Ionic droplet‐based interfacial iontronic sensing structure. Reproduced with permission.^[^ ^15^ ^]^ Copyright 2014, Royal Society of Chemistry. d) Ionic gel‐based interfacial iontronic sensing structure. Reproduced with permission.^[^ ^61^ ^]^ Copyright 2018, WILEY‐VCH. e) Ionic fabric‐based interfacial iontronic sensing structure. Reproduced with permission.^[^ ^13b^ ^]^ Copyright 2017, WILEY‐VCH.

Interfacial iontronic pressure sensing relies on the formation of an EDL at the electrolyte‐electrode interface.^[^ ^12^ ^]^ When pressure is applied, ions from the electrolyte redistribute near the electrode surface, forming a highly responsive capacitive layer that rapidly adjusts to external stimuli. The EDL consists of two distinct regions: the Helmholtz layer, where solvent molecules and counter‐ions are tightly bound to the electrode surface, and the diffuse layer, where ions are more loosely distributed to balance the electrode's surface charge (**Figure** **2**a).^[^ ^32^ ^]^ The total capacitance (*C*_EDL_) is represented by two interfacial capacitors in series:

(1) $$C_{\mathrm{EDL}}={\left(\frac{1}{C_{H}}+\frac{1}{C_{D}}\right)}^{-1}\;$$

here, *C*_H_ represents the Helmholtz layer capacitance and *C*_D_ denotes the diffuse layer capacitance. In the series configuration, the total capacitance (*C*_EDL_) is lower than either individual capacitance, primarily determined by the smaller of the two. *C*_H_ and *C*_D_ are influenced by several physical parameters, including the dielectric constant (*ε*), Helmholtz layer thickness (*d*), ionic species and concentrations (*C*), surface potential (*φ*), and temperature (*T*). The overall capacitance (*C*_EDL_) can also be expressed as:

(2) $$C_{\mathrm{EDL}}=\eta_{A}\;\cdot \phi \left(d,\varepsilon ,C,\phi ,T\right)\cdot A=UAC\cdot A$$

here, *η*_A​_ denotes the roughness factor, *ϕ*(*d,ϵ,C,ϕ,T*) represents a complex function of the listed parameters, and *A* is the electrode‐electrolyte contact area. Notably, the product of *η*_A​_ and *ϕ*(*d,ϵ,C,ϕ,T*) defines the EDL capacitance per unit area, termed UAC. In 2011, Pan et al.^[^ ^12^ ^]^ first applied this effect in interfacial capacitive sensing by demonstrating a droplet‐based pressure sensor with EDL's supercapacitive properties. This sensor, based on an ionic liquid droplet on a modified electrode, achieved ultrahigh capacitance in the range of several µF cm^−2^, far exceeding conventional capacitive sensors with capacitances in the tens to hundreds of pF cm^−2^.^[^ ^11^, ^33^ ^]^

Equation (2) shows that the EDL capacitance strongly depends on the electrode‐ion layer contact area, with the UAC governed by material and microstructure properties. Thus, micro‐structured sensing layers are crucial for enhancing the performance of EDL pressure sensors. When pressure is applied, these microstructures deform, which increases the contact area between the electrode and electrolyte and causes significant capacitance changes. For instance, in hemispherical microstructures (Figure 2b),^[^ ^34^ ^]^ Hertzian contact mechanics describe the relationship between contact area (*A*) and applied pressure (*P*):^[^ ^35^ ^]^

(3) $$A\propto P^{2/3}$$

This relationship demonstrates that the contact area increases nonlinearly with applied pressure, amplifying capacitance responses at low pressures. However, these nonlinear effects may lead to inconsistencies in sensor output. Precise microstructural designs, such as optimizing geometry and material composition, can mitigate these limitations and improve the linearity of the contact area‐pressure relationship across a wide pressure range. In addition to increasing contact area, microstructures enhance the electric field distribution at the electrode‐electrolyte interface,^[^ ^36^ ^]^ especially near sharp edges and tips where field intensification occurs. Such field enhancement promotes ion accumulation in the Helmholtz layer, thereby increasing overall capacitance. Furthermore, the integration of hierarchical microstructures with nano‐ and microscale features enhances the EDL effect. At low pressures, nanoscale features deform easily, increasing contact area and sensitivity. Microscale features maintain linear response at higher pressures, preventing saturation and ensuring stability. This multiscale design strategy optimizes the trade‐off between sensitivity and stability across a broad pressure range.

In addition to high sensitivity, these sensors exhibit fast response times due to rapid ionic rearrangement within the electrolytes. The high ionic mobility allows the sensors to respond rapidly to external stimuli. Moreover, the supercapacitor nature of the EDL, with high charge storage capability, stabilizes charge distribution and mitigates electromagnetic interference.^[^ ^12^, ^37^ ^]^

### 2.2 Material Strategies for Enhanced EDL Performance

The performance of EDL pressure sensors relies critically on the precise selection of electrode and ionic membrane materials. These materials are essential for optimizing charge transport, stabilizing EDL formation, and ensuring mechanical compliance under varying conditions.

#### 2.2.1 Electrode Materials

Electrode materials enable efficient charge transport and stabilize EDL formation at the electrode/electrolyte interface. Metals such as Au, Ag, and indium tin oxide (ITO), offer high electrical conductivity and electrochemical stability, making them suitable for high‐sensitivity applications.^[^ ^14^, ^38^ ^]^ However, their intrinsic brittleness restricts their applicability in stretchable devices. To address this limitation, Ag nanowires embedded in flexible matrices and liquid metal electrodes have improved flexibility and transparency, enhancing their suitability for wearable sensors.^[^ ^39^ ^]^ Nevertheless, their high cost and fabrication complexity remain significant challenges.

In contrast, carbon‐based materials such as graphene and carbon nanotubes (CNTs) stand out for their excellent conductivity, flexibility, and large surface area.^[^ ^40^ ^]^ When combined with flexible substrates, they form conductive networks that ensure stable EDL performance. However, for applications demanding strong adhesion to flexible substrates, conductive polymers like poly(3,4‐ethylenedioxythiophene):poly(styrenesulfonate) (PEDOT: PSS) and polypyrrole (PPy) are preferred for their adhesive properties and ease of processing.^[^ ^41^ ^]^ Additives like poly(vinyl alcohol) (PVA) or Co₃O₄ improve their mechanical properties and adhesion,^[^ ^42^ ^]^ making them ideal for applications requiring large deformation tolerance.

While materials like metals, carbon, and polymers offer distinct advantages, hybrid designs effectively address their limitations. For example, carbon‐based hybrids like carbon cloth@MnOx/MoS₂ integrate high conductivity and electrochemical activity.^[^ ^43^ ^]^ Fabric‐based composites like polydopamine (PDA)/MXene/stearic acid (STA) hybrids^[^ ^44^ ^]^ provide both breathability and durability. Polymer‐based composites, such as PDMS/SiO₂/silver nanoparticles,^[^ ^45^ ^]^ TiO₂/thermoplastic polyurethane (TPU)/silver nanowires (AgNWs),^[^ ^46^ ^]^ and PDMS‐CNT/Au,^[^ ^30^ ^]^ combine polymer elasticity with nanofiller conductivity, ensuring reliable performance under mechanical stress.

Advanced materials, such as nanocomposites and sustainable substrates, improve EDL sensor design by enhancing mechanical flexibility, sensitivity, and environmental compatibility. Nanocomposites like polyethylene terephthalate (PET)/Au/MXene enhance ion adsorption and interfacial stability.^[^ ^17^ ^]^ Biocompatible designs, such as silk fibroin (SF)/Au films,^[^ ^47^ ^]^ and biodegradable electrodes, like paper/Cu composites,^[^ ^34^ ^]^ enhance mechanical flexibility environmental compatibility. These features make them well‐suited for wearable and implantable devices.

#### 2.2.2 Ionic Membrane Materials

In EDL‐based flexible pressure sensors, ionic membranes serve as efficient ion conductors, facilitating rapid ion migration, stable ionic conductivity, and the formation of EDL capacitors with electrodes.^[^ ^48^ ^]^ These materials are categorized into three types: key ionic materials, functionalized ionic materials, and natural materials.

##### Key Ionic Materials

Key ionic materials, including ionic liquids (ILs) and ion gels, are favored for their high ionic conductivity and electrochemical stability. Pure ILs like 1‐ethyl‐3‐methylimidazolium tricyanomethanide ([EMIM][TCM]) and 1‐ethyl‐3‐methylimidazolium bis(trifluoromethylsulfonyl)imide ([EMIM][TFSI]) exhibit distinct advantages.^[^ ^24^, ^37^, ^49^ ^]^ [EMIM][TCM] facilitates rapid ion transport owing to its low viscosity, and [EMIM][TFSI] offers chemical stability in fluctuating environments. However, their fluid nature causes leakage and mechanical instability, limiting practical applications.^[^ ^49a^ ^]^ To address these issues, ion gels, formed by embedding ILs into polymer matrices, maintain the high conductivity of ILs while improving mechanical stability.

Fluoropolymer‐based ion gels, such as poly(vinylidene fluoride‐co‐hexafluoropropylene) (PVDF‐HFP) blended with [EMIM][TFSI],^[^ ^20^, ^40^, ^43^, ^49^ ^]^ exhibit robust dielectric properties and durability, making them ideal for high‐capacitance applications under harsh conditions. Acrylate‐based gels, like [EMIM][TFSI] blended with poly(ethylene glycol) diacrylate (PEGDA),^[^ ^34^ ^]^ leverage PEGDA's flexibility and rapid UV‐curing to form stretchable, mechanically durable structures. Elastomer‐based gels, such as poly([BMIM][SPA]‐co‐methyl acrylate) (P([BMIM][SPA]‐co‐MA))/hexanediol diacrylate (HDDA),^[^ ^50^ ^]^ employ covalent crosslinking to enhance elastic recovery, suitable for dynamic applications with repeated deformation.

Despite their benefits, synthetic ion gels face environmental and biocompatibility challenges, prompting interest in aqueous polymer‐based gels. Hydrophilic polymers like PVA and polyacrylamide (PAAm) form hydrogen‐bonded networks that provide mechanical stability and ionic transport efficiency.^[^ ^17^, ^51^ ^]^ For example, PVA/H₃PO₄ demonstrates high proton conductivity,^[^ ^51b^ ^]^ whereas PVA/NaCl/Glycerol improves flexibility and resilience at low temperatures.^[^ ^52^ ^]^ PAAm‐based gels, such as PAAm/NaCl,^[^ ^52^ ^]^ are particularly suitable for bio‐interfacing and transparent electronics due to their transparency and elasticity. Their hydrophilic nature ensures stable ionic transport under moderate humidity conditions, essential for consistent sensor performance.

##### Functionalized Ionic Materials

Functionalized ionic materials leverage surface modifications, nanocomposites, and tailored functional groups to enhance ionic conductivity, mechanical flexibility, and electrochemical stability. These materials are classified into four types: ionic liquid‐impregnated composites, modified ionic liquid composites, functionalized nanocomposites, and multifunctional ionic composites.

Ionic liquid‐impregnated composites enhance the electrochemical properties of flexible substrates by leveraging the high ionic conductivity and stability of ILs like [EMIM][TFSI] and 1‐butyl‐3‐methylimidazolium hexafluorophosphate ([BMIM][PF_6_]). In textile and fiber‐based matrices, ILs fill porous structures to form continuous ion transport networks, minimizing interfacial resistance and enabling rapid ion migration. For instance, [BMIM][PF_6_] improves ion transport in cellulose fibers by forming uniform ionic pathways, optimizing conductivity,^[^ ^53^ ^]^ while electrospun TPU/1‐ethyl‐3‐methylimidazolium bis(trifluoromethylsulfonyl)amide) ([EMIM][NTf_2_]) fibers enhance sensitivity due to their large surface area and efficient ion mobility.^[^ ^46^ ^]^ In polymer matrices like PDMS and polyurethane (PU) sponges,^[^ ^14^, ^21^, ^54^ ^]^ IL impregnation imparts ionic conductivity and stabilizes transport channels, ensuring reliable performance under mechanical deformation.

Modified ionic liquid composites, designed through doping and electrospinning, enhance mechanical and electrochemical properties. Doped electrospun fibers, such as P(VDF‐HFP)/[EMIM][TFSI],^[^ ^13b^ ^]^ feature high surface area and interconnected nanoporous networks, facilitating uniform ion distribution and rapid migration. This architecture accelerates EDL formation, improving sensor responsiveness in high‐frequency dynamic pressure applications. In contrast, physically blended composites like TPU/[EMIM][TFSI] leverage strong hydrogen bonding and ionic interactions to create stabilized networks. This balance of ionic conductivity and mechanical flexibility makes them highly suitable for long‐term wearable devices under repeated deformation.^[^ ^55^ ^]^

Functionalized nanocomposites integrate IL‐polymer matrices with nanostructures like MXene, TiO_2_, and hexagonal boron nitride (*h*‐BN) to enhance sensitivity and durability. MXenes, with their high surface area and exceptional conductivity, facilitate ion adsorption and charge transfer, greatly enhancing sensor sensitivity.^[^ ^56^ ^]^ TiO₂ enhances mechanical strength while providing UV shielding and antibacterial properties, making it suitable for outdoor and biomedical sensors.^[^ ^46^ ^]^ Dielectric fillers like *h*‐BN modulate the composite's dielectric constant and flexibility, ensuring stable and precise pressure responses.^[^ ^16^ ^]^ The synergistic effects of these nanostructures enhance ion transport and mechanical stability, making them particularly effective for dynamic pressure sensing and wearable devices.

Multifunctional ionic composites, incorporating additives like lithium chloride (LiCl) or deep eutectic solvents (DES), overcome challenges posed by low temperatures and humidity. For instance, LiCl/2‐hydroxyethyl acrylate (HEA)/ethylene oxide‐ethylene oxide ethyl acrylate (EOEOEA) composites exhibit anti‐freezing and self‐healing properties, suitable for extreme environments.^[^ ^57^ ^]^ Additionally, systems like poly(acrylic acid)‐tannic acid/cellulose nanocrystals‐choline chloride (PAA‐TA/CNC‐ChCl) offer self‐adhesion and flexibility, enabling their use in conformal wearable applications.^[^ ^58^ ^]^

##### Natural Materials

Natural materials provide a sustainable alternative to ionic membranes in EDL pressure sensors. Plant‐based materials, such as rose petals,^[^ ^26^ ^]^ with hierarchical microstructures, improve sensitivity and stability by facilitating efficient ion transport and EDL formation. Human skin, as a natural iontronic interface, leverages its intrinsic ionic conductivity and flexibility to form effective EDLs, enabling high‐resolution sensing with minimal fabrication complexity.^[^ ^59^ ^]^ These natural materials contribute to the development of eco‐friendly, high‐performance sensors, particularly for real‐time health monitoring and wearable applications.

In summary, ionic membrane materials for EDL pressure sensors range from liquid ionic materials to advanced solid‐state systems. Pure ionic liquids exhibit excellent ionic conductivity but often suffer from leakage and evaporation, which limit their stability. In comparison, solid‐state materials like polymer‐based ion gels, aqueous polyelectrolytes, and multifunctional composites offer superior mechanical stability, electrochemical performance, and durability (Figure 2c–e). On the other hand, natural materials, such as rose petals and human skin, provide eco‐friendly and biocompatible alternatives, presenting promising solutions for wearable electronics and health monitoring applications.

## 3 Structural Engineering of Sensing Layers

Structural engineering of sensing layers underpins the performance enhancement of flexible EDL pressure sensors. Nanoscale to multiscale hierarchical morphologies critically influence the sensing capabilities of these devices. This section categorizes structural design strategies, linking their impact on sensor performance (Section 4) with the fabrication methods enabling these designs (Section 5).

### 3.1 Nanoscale Structural Design

Nanoscale structural optimization is pivotal for enhancing EDL sensor performance, particularly through functional material strategies and structural design approaches. Functional materials target nanomaterials with high electrochemical activity, while structural designs focus on geometric manipulation to improve surface interactions and mechanical stability.

#### 3.1.1 Functional Material Strategies

Functional material strategies utilize advanced nanomaterials to enhance iontronic properties by improving ion transport and charge storage. Key approaches include exploiting ion pump effects,^[^ ^16^, ^24^, ^56^, ^62^ ^]^ and incorporating pseudocapacitive materials.^[^ ^17^, ^40^, ^43^, ^63^ ^]^

**Figure 3:**
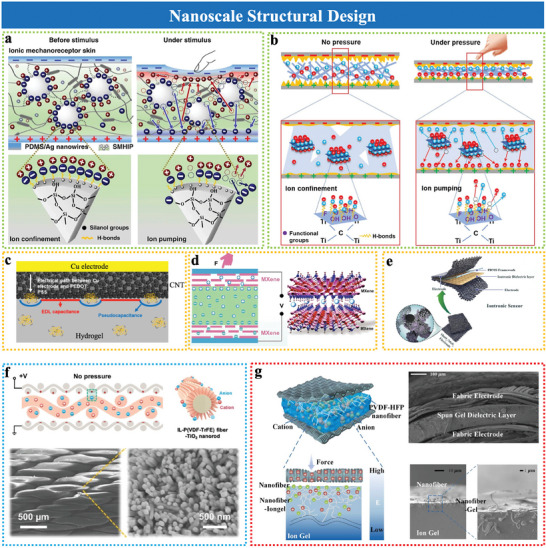
Sensing materials with nanoscale structural design. a) Schematic of the sensing mechanism based on the ion pump effect using IL/TPU/silica composite. Reproduced with permission.^[^ ^24^ ^]^ Copyright 2019, Springer Nature. b) Ionic nanofibrous membrane (IL‐MXene‐PVA composite). Reproduced with permission.^[^ ^56^ ^]^ Copyright 2021, American Chemical Society. c) Pseudocapacitive materials‐based PEDOT: PSS/vinyl silica nanoparticle (VSNP)‐PAAm iontronic film. Reproduced with permission.^[^ ^40a^ ^]^ Copyright 2022, American Chemical Society. d) MXene electrode. Reproduced with permission.^[^ ^17^ ^]^ Copyright 2021, Springer Nature. e) MnO_x_/MoS_2_ electrode. Reproduced with permission.^[^ ^43^ ^]^ Copyright 2024, Elsevier. f) Growth of TiO_2_ nanorods on P(VDF‐TrFE) fibers, with corresponding SEM images. Reproduced with permission.^[^ ^19^ ^]^ Copyright 2023, Wiley‐VCH. g) Nanofiber‐ionogel sensor with gradient stiffness design, SEM cross‐section and surface micrograph. Reproduced with permission.^[^ ^18^ ^]^ Copyright 2024, Elsevier.

Dynamic ion transport is critical for modulating the EDL under external stimuli. Tailored nanoparticles and nanosheets, with high surface areas and customized ionic environments, enhance ion confinement and charge redistribution.^[^ ^16^, ^24^, ^56^, ^62^ ^]^ For example, Amoli et al.^[^ ^24^ ^]^ developed an ionic pressure sensor by integrating hydrogen‐bonded ion pairs onto SiO_2_ microspheres within a TPU matrix (**Figure** **3**a). The reversible interactions between ionic pairs and the SiO_2_ surface improved structural integrity and ion management, enabling stable EDL formation and high sensitivity across a wide pressure range. Similarly, MXene nanosheets prepared via vacuum filtration^[^ ^56^ ^]^ and *h*‐BN^[^ ^16^ ^]^ leverage their layered structures and high aspect ratios^[^ ^64^ ^]^ to enhance surface ion interactions and charge distribution.^[^ ^65^ ^]^ For example, Sharma et al.^[^ ^56^ ^]^ designed a hybrid ionic nanofibrous membrane by incorporating MXene nanosheets and lithium sulfonamide ionic salts into a PVA elastomer matrix (Figure 3b). The functional layer on the MXene surface traps ions via hydrogen bonding, reducing initial capacitance by inhibiting EDL formation at the electrode/electrolyte interface. Under external pressure, the ion pumping mechanism induces the formation of a thick EDL, leading to substantial capacitance variations and enhanced pressure sensitivity. Despite these advancements, challenges like batch‐to‐batch variability in MXene processing and limited nanosheet dispersion remain, necessitating further optimization of fabrication methods.

Pseudocapacitive materials, such as graphene,^[^ ^40^, ^66^ ^]^ MXene,^[^ ^63^, ^67^ ^]^ PEDOT,^[^ ^68^ ^]^ MoS_2_,^[^ ^63a^ ^]^ and MnO_x_/MoS_2_^[^ ^43^ ^]^ are known for their large surface areas, electrical conductivity, chemical stability, which enable efficient ion intercalation and charge storage. For instance, a hybrid iontronic sensor integrates pseudocapacitive PEDOT into the iontronic film, alongside EDL‐capacitive carbon nanotubes, significantly enhancing capacitance and sensitivity (Figure 3c).^[^ ^68^ ^]^ Similarly, MXene electrodes achieve ultrahigh sensitivity (45 000 kPa^−1^) and a broad sensing (20 Pa to 1.4 MPa) due to ion intercalation in their layered structure (Figure 3d). However, MXene's oxidation susceptibility limits its long‐term stability, necessitating further surface modification. MoS_2_, a conductive and chemically stable 2D material, exhibits strong pseudocapacitive behavior.^[^ ^69^ ^]^ Xu et al.^[^ ^63a^ ^]^ demonstrated significant sensitivity enhancement with a monolayer MoS_2_ electrode, showcasing its strong pseudocapacitive behavior. Additionally, the MnOx/MoS_2_ composite electrode^[^ ^43^ ^]^ integrates the large surface area of MnO_x_ with the conductivity of MoS_2_, further enhancing charge storage, mechanical robustnes, and electrochemical stability (Figure 3e). Future efforts should prioritize scalable manufacturing techniques, such as CVD or electrochemical deposition, to ensure consistent performance and broader applicability.

These engineered nanomaterials, with their unique morphologies and expansive surface areas, provide a foundation for advanced EDL sensing and a basis for understanding how nanoscale features influence sensor performance, complementing geometric optimization approaches.

#### 3.1.2 Structural Design Strategies

Structural design strategies optimize nanoscale architectures to enhance mechanical integrity and surface ion interactions. Key approaches include nanorods and gradient stiffness designs, achieved through precise manufacturing tailored to specific structural requirements.

TiO₂ nanorods are synthesized via hydrothermal growth on flexible substrates, such as poly(vinylidene fluoride‐co‐trifluoroethylene) (P(VDF‐TrFE)) fibers, enabling precise control of alignment and aspect ratio (Figure 3f).^[^ ^19^ ^]^ Adjusting parameters such as precursor concentration and temperature forms vertically aligned nanorods, which maximize the effective contact area and improve interfacial ion dynamics. These structures also reduce initial contact area with electrodes, amplifying sensitivity and preserving flexibility. However, consistent alignment and scalability remains challenging, requiring advancements in template‐assisted synthesis.

Gradient stiffness designs achieve mechanical transitions in the sensing layer by integrating electrospun PVDF‐HFP nanofibers into an ionogel matrix (Figure 3g).^[^ ^18^ ^]^ Electrospinning conditions, such as polymer concentration and applied voltage, are optimized to control fiber stiffness and alignment, enabling precise gradient architectures. This design enhances compressive strength, accelerates ion migration, and mitigates stress concentration, thereby improving the sensor's pressure detection range, stability, and durability under repeated deformation. Despite these advantages, large‐scale production remains challenging, requiring multi‐jet electrospinning systems for uniformity and scalability.

These structural design strategies leverage nanoscale architectures to enhance ion transport, mechanical strength, and sensing reliability, addressing critical challenges in EDL sensors and paving the way for scalable applications.  

### 3.2 Microscale Structural Design

Microscale structural designs in EDL pressure sensors including microroughness, internal porosity, and multiscale hierarchical structures, offer distinct performance advantages.

#### 3.2.1 Micro‐roughness Structure

Micro‐roughness structures of sensing materials are categorized into micro‐geometries,^[^ ^20^, ^51^, ^61^, ^70^ ^]^ bionic surfaces,^[^ ^20^, ^27^, ^44^, ^71^ ^]^ and wrinkled roughness types.^[^ ^72^ ^]^ These microstructures increase material deformability and maximize contact area at the electrode‐dielectric interface, leading to substantial improvements in capacitance change and sensitivity.

**Figure 4:**
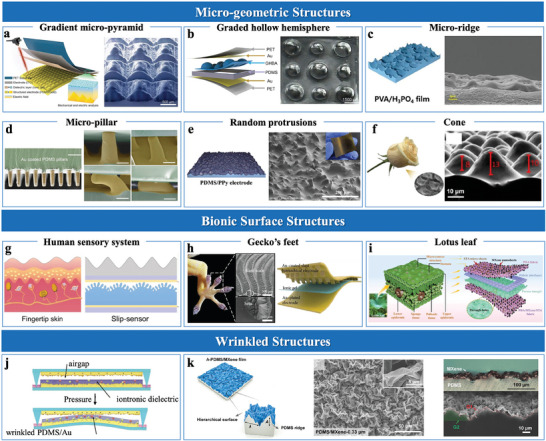
Sensing materials with micro‐rough structures. a) Gradient micro‐pyramid structure. Reproduced with permission.^[^ ^20b^ ^]^ Copyright 2023, Springer Nature. b) Graded hollow hemisphere. Reproduced with permission.^[^ ^51b^ ^]^ Copyright 2024, WILEY‐VCH. c) Micro‐ridge structure. Reproduced with permission.^[^ ^45^ ^]^ Copyright 2022, Wiley‐VCH. d) Micro‐pillar structure. Reproduced with permission.^[^ ^59^ ^]^ Copyright 2021, Springer Nature. e) Graded intra‐fillable structure. Reproduced with permission.^[^ ^70a^ ^]^ Copyright 2020, Springer Nature. f) Cone structure. Reproduced with permission.^[^ ^75^ ^]^ Copyright 2021, Springer Nature. g) Structure inspired by human sensory system. Reproduced with permission.^[^ ^20c^ ^]^ Copyright 2023, Springer Nature. h) Structure inspired by gecko's feet. Reproduced with permission.^[^ ^27^ ^]^ Copyright 2022, Yongsong Luo et al. i) Structure inspired by lotus leaf. Reproduced with permission.^[^ ^44^ ^]^ Copyright 2023, Elsevier. j) Wrinkled, stretchable PDMS/Au electrodes. Reproduced with permission.^[^ ^72b^ ^]^ Copyright 2022, Wiley‐VCH. k) Hierarchical PDMS/MXene film with a wrinkled MXene layer. Reproduced with permission.^[^ ^72a^ ^]^ Copyright 2023, Elsevier.

Micro‐geometric structures, such as pyramids,^[^ ^20^, ^38^, ^49^, ^51^ ^]^ effectively enhance capacitance variation by rapidly modulating contact areas under pressure. Xiong et al.^[^ ^49b^ ^]^ demonstrated that pyramidal microstructures with varying heights (11 and 30 µm) improved sensitivity from 0.05 to 4.82 kPa⁻¹, illustrating the importance of geometric precision in tuning performance. Recent advancements, such as CO₂ laser assisted fabrication, have enabled precise control over pyramid profiles and heights by adjusting laser power and scanning speed, offering a cost‐effective alternative to photolithography technique (**Figure** **4**a).^[^ ^20b^ ^]^ Hemispherical structures have also been widely adopted owing to their robust architecture and mechanical stability.^[^ ^34^, ^70^ ^]^ For instance, Shen et al.^[^ ^34^ ^]^ found that spherical caps with smaller contact angles generally exhibit higher sensitivity but are less deformable than pyramid shapes, limiting their sensitivity. To address this limitation, Ding et al.^[^ ^51b^ ^]^ developed a graded hollow ball arch microstructure to enhance compressibility and contact area variation (Figure 4b). This design improves deformation under pressure, enabling precise sensitivity across a wide pressure range. Additionally, Chen et al.^[^ ^45^ ^]^ proposed an arete‐like hierarchical architecture combing hemispherical stability with pyramidal contact dynamics to improve linearity and sensitivity (Figure 4c). Moreover, micropillar electrodes demonstrated adaptability to varying pressure conditions through a multi‐stage deformation process,^[^ ^59^, ^70^ ^]^ including initial contact, pre‐buckling, buckling, and post‐buckling. Zhu et al.^[^ ^59^ ^]^ demonstrated that high‐aspect‐ratio micropillar electrodes significantly enhanced contact area and sensitivity in skin‐electrode interfaces (Figure 4d). However, fabricating such microstructures involves complex processes (e.g., photolithography), which poses challenges in scalability. Simpler approaches, like using sandpaper templates, provide cost‐effective and efficient alternatives to conventional methods.^[^ ^41^, ^70^, ^73^ ^]^ Zheng et al.^[^ ^41a^ ^]^ developed random protrusion microstructures to enhance sensitivity by modulating electrode‐dielectric contact, improving compressibility, and broadening the pressure‐response range (Figure 4e). Additionally, natural templates, like plant leaves^[^ ^61^, ^74^ ^]^ and rose petals,^[^ ^75^ ^]^ effectively create surface microstructures. Liu et al.^[^ ^75^ ^]^ utilized rose petals as templates to form uniform cone‐like structures, balancing simplicity and scalability (Figure 4f).

Bionic microstructures inspired by nature, mimicking human skin and hair,^[^ ^20^, ^71^ ^]^ animal epidermis,^[^ ^27^, ^71^ ^]^ and plant leaf structures,^[^ ^44^, ^71^, ^74^ ^]^ effectively enhance sensor functionality through advanced microstructural morphologies. For instance, Bai et al.^[^ ^20c^ ^]^ developed an artificial sensory system inspired by human skin receptors, employing high‐precision 3D printing and PDMS molding to replicate the structural characteristics of slow adaptive and fast adaptive mechanoreceptors. This design enables high spatial and temporal resolution, supporting static pressure and dynamic vibration detection (Figure 4g). In addition, inspired by the hierarchical slant scales of gecko feet, Luo et al.^[^ ^27^ ^]^ utilized oblique lithography to fabricate slant hierarchical microstructures as electrodes interfacing with ionic gel layers. These structures minimize pressure resistance via bending deformation and expand the functional interface area, achieving ultra‐high sensitivity (Figure 4h). Moreover, mimicking the micro‐channels and through‐holes of lotus leaves, Sun et al.^[^ ^44^ ^]^ developed bioinspired surfaces by embedding hydrophobic STA micro‐sheets into the electrode surface and using a porous ionogel as the dielectric layer (Figure 4i). These structures enhance flexibility, breathability, and self‐cleaning capabilities, ensuring reliable functionality in both wet and dry environments.

Wrinkled micro‐rough structures offer a scalable and cost‐effective solution for EDL pressure sensor design.^[^ ^72^ ^]^ These structures increase surface area, enhance ion‐electrode interactions and mechanical adaptability, offering the flexibility and stretchability required for dynamic sensing applications. Rwei et al.^[^ ^72b^ ^]^ utilized heat‐shrinkable polymer templates to fabricate wrinkled and stretchable PDMS/Au electrodes, controlling the degree of wrinkling through heating temperature and polymer shrinkage rate. This design maintained stable electrical conductivity even under substantial mechanical strain (Figure 4j). Similarly, Chen et al.^[^ ^72a^ ^]^ fabricated conductive PDMS/MXene films with hierarchical wrinkles, enhancing surface area and mechanical adaptability (Figure 4k). These design approaches enhance the structural adaptability of EDL sensors, ensuring functionality under mechanical deformation.

#### 3.2.2 Internal Porous Structure

The sensitivity of EDL pressure sensors largely depends on the contact area at the electrode‐dielectric interface. Traditional surface microstructures are widely employed to maximize this contact area. However, these designs often harden under increased pressure, reducing adaptability. In contrast, porous materials, characterized by low stiffness and high compressibility, offer distinct advantage by accommodating deformation under load and maintaining enhanced contact performance.

**Figure 5:**
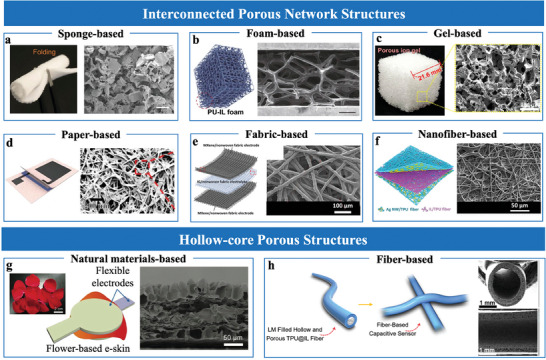
Sensing materials with internal porous structures. a) Sponge‐based structure. Reproduced with permission.^[^ ^21a^ ^]^ Copyright 2023, Wiley‐VCH. b) Foam‐based structure. Reproduced with permission.^[^ ^14^ ^]^ Copyright 2021, Springer Nature. c) Gel‐based structure. Reproduced with permission.^[^ ^21b^ ^]^ Copyright 2021, American Chemical Society. d) Paper‐based structure. Reproduced with permission.^[^ ^76b^ ^]^ Copyright 2019, WILEY‐VCH. e) Fabric‐based structure. Reproduced with permission.^[^ ^77^ ^]^ Copyright 2022, American Chemical Society. f) Nanofiber‐based structure. Reproduced with permission.^[^ ^78c^ ^]^ Copyright 2022, Elsevier. g) Hollow porous structure derived from natural materials. Reproduced with permission.^[^ ^26^ ^]^ Copyright 2018, WILEY‐VCH. h) Hollow ionogel fibers. Reproduced with permission.^[^ ^39b^ ^]^ Copyright 2022, Elsevier.

Porous materials with interconnected frameworks, such as sponges^[^ ^54^ ^]^ and foams,^[^ ^14^, ^62^ ^]^ are widely utilized as elastic skeletons in sensors for their porosity and flexibility. These structures are often impregnated with sensing materials. For instance, Wang et al.^[^ ^21a^ ^]^ used a sacrificial template method to fabricate a porous PDMS sponge, which was impregnated with ionic liquid to form an iontronic layer. The interconnected porous network enhanced the contact area under pressure, improving sensitivity (**Figure** **5**a). Similarly, Liu et al.^[^ ^14^ ^]^ employed open‐cell PU foams with high porosity and low modulus, allowing efficient ionic liquid integration through a one‐step soaking process (Figure 5b). The high porosity and deformability of these foams maximized contact area and responsiveness.

Gel‐based materials are inherently porous, naturally increasing contact area and enhancing adaptability under pressure, making them ideal for sensor design.^[^ ^48^ ^]^ For instance, Kwon et al.^[^ ^21b^ ^]^ developed porous ion gels using sugar cube templates (Figure 5c). A cross‐linked copolymer network poly (ethyl acrylate‐ran‐styrene‐randivinylbenzene) (PEA‐*r*‐PS‐*r*‐PDVB) was formed within the sugar template and dissolved after polymerization, creating a highly porous structure. The resulting structure was infused with ionic liquid ([EMIM][TFSI]), enhancing pressure response and electrochemical performance. The interconnected porous network increased the contact area under pressure, improving sensitivity and adaptability for EDL pressure sensor applications.

Paper‐based, fabric‐based, and nanofiber‐based sensing layers expanded the design possibilities of EDL pressure sensors, offering tailored solutions for diverse applications. Paper‐based materials provide eco‐friendly and cost‐effective alternatives, with sensing layers fabricated through methods like dip‐coating or handwriting.^[^ ^76^ ^]^ For instance, Pan et al.^[^ ^76b^ ^]^ introduced the first iontronic paper with integrated ionic and conductive patterns, creating an integrated platform for flexible sensing application (Figure 5d). These materials can be tailored for pressure/force sensing through simple manipulations. Fabric‐based and nanofiber‐based materials are particularly suited for wearable devices due to their lightness, breathability, and robustness. Commercial fabrics can be functionalized by immersing them in ionic liquids, such as [BMIM]PF_6_^[^ ^53^ ^]^ and [EMIM][TFSI],^[^ ^77^ ^]^ forming flexible, breathable sensing layers. The dip‐coating process ensures uniform ionic liquid distribution, enhancing ion transport pathways and mechanical adaptability under pressure. Nanofiber‐based materials, fabricated via electrospinning, combine high surface area with precise control over fiber diameter and distribution. Ionic materials are incorporated into nanofibers through two approaches: direct blending into the electrospinning solution,^[^ ^13^, ^40^, ^78^ ^]^ or post‐treatment via dip‐coating.^[^ ^46^, ^47^ ^]^ For example, Wang et al.^[^ ^78c^ ^]^ developed TPU nanofibers with enhanced flexibility and breathability by controlling fiber alignment and diameter (Figure 5f). Additionally, advancements in materials synthesis have enabled durable, water‐resistant devices. Sun et al.^[^ ^78a^ ^]^ demonstrated hydrophobic poly(ionic liquid)‐based nanofibrous that maintained stability after repeated water washing.

Hollow materials with high compression resilience are widely used in EDL pressure sensors for their enhanced contact area and deformation capabilities, leading to superior sensitivity. For instance, Guo et al.^[^ ^26^ ^]^ used critical point‐dried materials (e.g., rose petals, rose leaves, and *Acacia Mill* leaves) as dielectric layers with foam‐like hollow structures (Figure 5g). These structures exhibited high compressibility and functioned as metamaterials with surface microstructures that further amplified sensitivity. In addition, Zhu et al.^[^ ^39b^ ^]^ developed fiber‐based iontronic sensors using hollow and porous TPU/IL ionogel fibers filled with liquid metal electrodes (Figure 5h). The integration of liquid metal enabled substantial interfacial area changes during pressure application, significantly enhancing compressibility and sensitivity. This design further extended the pressure sensing range while maintaining robust mechanical adaptability, highlighting the potential of combining hollow architectures with conductive fillers for advanced EDL sensors.

#### 3.2.3 Multiscale Hierarchical Structure

Multiscale hierarchical structures in EDL pressure sensors represent innovative approaches for enhancing sensitivity, operational range, and overall performance. These structures are categorized into multi‐level microstructures,^[^ ^22^, ^79^ ^]^ stacked microstructures,^[^ ^80^ ^]^ and hierarchical interlocking structures,^[^ ^25^ ^]^ each optimized for mechanical adaptability and pressure response.

**Figure 6:**
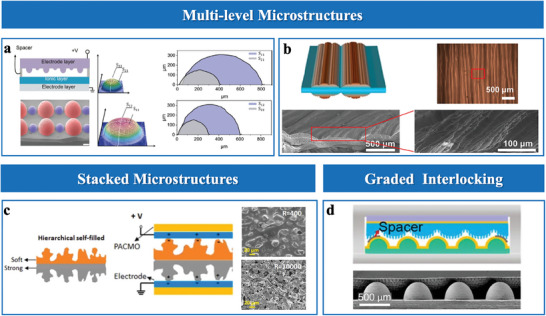
Sensing materials with multiscale hierarchical microstructures. a) Graded microstructures with semi‐elliptical convexities. Reproduced with permission.^[^ ^79c^ ^]^ Copyright 2024, Wiley‐VCH. b) Multiple graded wrinkle constructions. Reproduced with permission.^[^ ^79b^ ^]^ Copyright 2024, American Chemical Society. c) Multilayer self‐filled microstructure. Reproduced with permission.^[^ ^82^ ^]^ Copyright 2021, American Chemical Society. d) Graded interlocks. Reproduced with permission.^[^ ^25^ ^]^ Copyright 2022, American Chemical Society.

Multi‐level microstructures incorporate multiple hierarchical levels of structural features that synergistically enhance sensor functionalities.^[^ ^81^ ^]^ Wang et al.^[^ ^79c^ ^]^ developed graded microstructures with semi‐elliptical convexities on the electrode layer (**Figure** **6**a). These features progressively increase the contact area under pressure, enhancing sensitivity and sensing range. Additionally, Chen et al.^[^ ^79b^ ^]^ employed repeated stretching to induce multi‐level wrinkle structures (Figure 6b). Larger wrinkles broaden the pressure response range, while smaller wrinkles maximize sensitivity by precisely modulating contact area.

Stacked microstructures are characterized by multilayer architectures that enhance structural compressibility, optimize stress distribution, and enable adaptable pressure responses in EDL sensors. These designs integrate complementary features, such as dual‐sided microstructures or sequentially responsive layers, to balance sensitivity and operational range. For instance, Zammali et al.^[^ ^82^ ^]^ developed a multilayer self‐filled iontronic sensor, where soft upper and stiff lower layers engage sequentially under varying pressures, achieving continuous compressibility and a broad sensing range (Figure 6c). Meanwhile, Xiao et al.^[^ ^80^ ^]^ utilized dual‐sided microstructures to synchronize surface interactions, improving stress distribution and ensuring consistent performance across different pressures.

Graded interlocking structures, integrating features across multiple scales, have proven effective in enhancing sensor compressibility and uniform stress distribution. These structures are typically fabricated through templating or micro‐patterning, enabling precise integration of larger structural elements, such as hemispheres, with finer microstructures like pillars (Figure 6d).^[^ ^25^ ^]^ Larger elements provide mechanical stability and load distribution, while the finer features enhance surface interactions and deformation adaptability. This synergistic design mitigates stiffening effects, expanding the linear pressure‐response range, and improving sensitivity. The versatility and adaptability of graded interlocking architectures highlight their potential solutions to the dual challenges of sensitivity and operational range.

### 3.3 AI‐Enabled Material and Structural Design

Nanoscale and microscale structural optimizations are pivotal in advancing EDL‐based pressure sensors. Traditional strategies, including finite element modeling, functional material enhancements, and geometric configurations, have significantly improved sensor performance. However, these methods often involve resource‐intensive iterations to address the complex interplay between material properties and structural geometries. Artificial Intelligence‐driven design approaches provide transformative solutions though data‐driven modeling, inverse design, and generative techniques, enabling efficient exploration of material and structural configurations.^[^ ^83^ ^]^ These methods reduce development cycles and address multi‐objective optimization challenges, accelerating the design of high‐performance EDL sensors.

#### 3.3.1 AI‐Driven Materials Optimization

Material selection directly affects EDL sensor performance by enhancing charge density and ionic transport. Conventional methods, like finite element‐based simulations and experimental screening, are effective but time‐consuming. They are also limited in resolving trade‐offs between ionic conductivity, mechanical flexibility, and stability. AI‐driven methodologies, successful in other fields,^[^ ^84^ ^]^ address these challenges through predictive modeling, design space exploration, and generative algorithms.

Advanced models such as the Atomistic Line Graph Neural Network (ALIGNN) predict atomic‐level properties, such as formation energy and elastic moduli, with high accuracy and computational efficiency.^[^ ^84g^ ^]^ ALIGNN optimizes materials like MXenes, known for their charge storage and chemical stability, by identifying those with enhanced electronic properties and mechanical durability. Similarly, bayesian optimization (BO) efficiently explores high‐dimensional design spaces to balance complex performance requirements. For example, BOWSR (Bayesian Optimization with Symmetry Relaxation) enables high‐throughput screening of material databases, facilitating the discovery of ion‐conductive membranes and electrode materials with optimal combinations of conductivity, flexibility, and stability.^[^ ^84d^ ^]^

Generative models, such as Generative Adversarial Networks (GANs) and Variational Autoencoders (VAEs), provide additional capabilities by autonomously generating novel material configurations.^[^ ^84e^ ^]^ For instance, GANs predict crystal structures, while VAEs identify materials with improved elasticity and flexibility. These models can be adapted to design advanced electrode or membrane materials for EDL sensors. Furthermore, active learning and data augmentation address data scarcity by prioritizing informative datasets and generating synthetic data, enhancing model training efficiency and design outcomes.^[^ ^85^ ^]^

#### 3.3.2 AI‐Assisted Microstructure Optimization

Optimizing microstructural geometries is critical for enhancing the performance of EDL flexible pressure sensors. Traditional approaches, such as finite element analysis (FEA) and topology optimization, face limitations like high computational costs, nonlinear material behaviors, and difficulties in modeling complex multiscale interactions. AI‐driven structural design enables rapid exploration of advanced configurations,^[^ ^86^ ^]^ optimizing critical properties such as sensitivity, linearity, and stability.

For example, Guo et al.^[^ ^86d^ ^]^ proposed a data‐driven inverse design framework for optimizing microstructures in EDL pressure sensors. This method combines jumping‐selection and surrogate modeling to efficiently explore high‐dimensional design spaces, identifying promising microstructures through predictive scoring. This framework reduces computational overhead and dataset requirements while achieving high design accuracy. A notable outcome was the development of microstructures with a near‐perfect linear response (R^2^ = 0.999) and higher proportion of viable configurations. These optimized geometries demonstrated superior performance in sensitivity, stability, and linearity, showcasing the ability of inverse design to surpass traditional trial‐and‐error methods. Additionally, the scalability of this approach provides a robust foundation for exploring multiscale microstructure optimization.

Knowledge‐graph‐based learning frameworks further enhance AI‐driven design by integrating experimental and theoretical data to identify correlations between structural features and performance metrics.^[^ ^86e^ ^]^ Although primarily applied in strain sensors, these frameworks hole potential for extension to EDL sensors. For instance, knowledge graphs could guide the design of electrode surfaces with tailored roughness or porosity to maximize capacitance or optimize hierarchical membrane structures to balance mechanical flexibility and ionic conductivity. By addressing data scarcity and enabling targeted design exploration, knowledge graphs are invaluable for developing innovative microstructural architectures in EDL sensors.

In conclusion, AI‐driven methodologies in material selection and structural optimization provide transformative advancements for EDL pressure sensors. Traditional strategies, such as nanoscale material design and hierarchical microstructures, have improved sensor performance but struggle to address complex trade‐offs and multi‐objective requirements. Techniques like graph neural networks, bayesian optimization, and generative models accelerate the discovery of high‐performance materials and optimized microstructures, driving breakthroughs in sensitivity, stability, and scalability. Leveraging AI's predictive accuracy and efficiency, researchers can integrate these methods with existing strategies to develop adaptive sensors for diverse applications. **Table** **1** provides a summary and comparison of EDL pressure sensors with different structures and materials.

**Table 1:** Summary and comparison of EDL pressure sensors by structural designs and ion membrane materials.

Type	Ion membrane materials	Sensitivity	Linearity [R^2^]	Working range	Response time	Limit of detection	Durability	Ref.
Functional materials	TPU/SiO_2_/[EMIM][TFSI]	48.1kPa^−1^	‐	135 kPa	60 ms	‐	500 (12.1 kPa)	[24]
	MXene/PVA/[Li][TFSI]	5.5 kPa^−1^	‐	250 kPa	70.4 ms	2 Pa	20 000(45 kPa)	[56]
	TPU/[EMIM][TFSI]/*h*‐BN	1307.7 kPa^−1^	0.983	450 kPa	15 ms	50 Pa	5000 (400 kPa)	[16]
	PEDOT:PSS/VSNP‐PAAm/CNT	301.5 kPa^−1^	‐	63.3kPa	197 ms	25 Pa	3000	[68]
	P(VDF‐HFP)/[EMIM][TFSI]	12.8 kPa^−1^	‐	1000 kPa	20 ms	2.5 Pa	10 000 (1 MPa)	[40a]
	PVA‐KOH	46 730 kPa^−1^	0.99	1.4 MPa	98 ms	20 Pa	10 000 (510 kPa)	[17]
	PVA/H_3_PO_4_	89.75 kPa^−1^	0.956	722.2 kPa	3 ms	‐	5000(138.9 kPa)	[63a]
	P(VDF‐HFP)/[EMIM][TFSI]	58 853 kPa^−1^	‐	1000 kPa	1.95 ms	8.6 mg	10 000(195.2 kPa)	[43]
Nanoscale structures	[EMIM][TFSI]/P(VDF‐TrFE)/TiO_2_	15.06 kPa^−1^	‐	200 kPa	5.6ms	2 Pa	18 000 (22 kPa)	[19]
	P(VDF‐HFP) nanofiber/PVA gel	10 159.69kPa^−1^	‐	1000 kPa	120 ms	50mg	5000 (100 kPa)	[18]
Micro‐roughness Structure	P(VDF‐HFP)/[EMIM][TFSI]	4.82 kPa^−1^	0.995	38 kPa	35 ms	40Pa	5000 (20 kPa)	[49b]
	P(VDF‐HFP)/[EMIM][TFSI]	33.7 kPa^−1^	0.99	1700 kPa	6ms	0.36Pa	4500 (800 kPa)	[20b]
	P(VDF‐HFP)/[EMIM][TFSA]	41.64 kPa^−1^	‐	50 kPa	21ms	<1Pa	5000 (3 kPa)	[38b]
	ATMP‐PVA	3224.2 kPa^−1^	0.994	0‐100 kPa	6ms	0.5Pa	200 (5 kPa)	[51a]
	TPU/[EMIM][TFSI]	1.4 nF kPa^−1^	‐	>10 kPa	47 ms	100Pa	200 (30 kPa)	[70b]
	[EMIM][TFSI]/PEGDA/HOMPP	100 nF kPa^−1^	‐	100kPa	‐	‐	1000 (55 kPa)	[34]
	PVA/H_3_PO_4_	10 420.8 kPa^−1^	0.99	300 kPa	40ms	0.2Pa	1800 (300 kPa)	[51b]
	PVA/H_3_PO_4_	20.98 kPa^−1^	0.9921	0‐37.5 kPa	30ms	0.67 Pa	6000 (11.7 kPa)	[45]
	P(VDF‐HFP)/[EMIM][TFSI]	33.16 kPa^−1^	0.999	176kPa	9 ms	0.9 Pa	6000 (80 kPa)	[70c]
	Human skin	11.8 kPa^−1^	‐	15 kPa	15 ms	0.2Pa	5000 (5 kPa)	[59]
	P(VDF‐HFP)/[EMIM][TFSI])	131.5 kPa^−1^	0.924	32.35kPa	43 ms	1.12 Pa	7000 (470 Pa)	[73]
	P(VDF‐HFP)/[EMIM][TFSI]	145.45 kPa^−1^	‐	50 kPa	44 ms	0.4Pa	6200 (0.1 kPa)	[87]
	P(VDF‐TrFE‐CFE) P(VDF‐HFP)/[EMIM][TFSA]	26.6 kPa^−1^	‐	100 kPa	48ms	2.88 Pa	8000 (2.2 kPa)	[41a]
	PVA/H_3_PO_4_	3302.9 kPa^−1^	‐	360 kPa	9 ms	0.08Pa	5000 (300 kPa)	[70a]
	P(VDF‐HFP)/[EMIM][TFSI]	54.32 kPa^−1^	‐	115kPa	29 ms	0.1 Pa	5400 (0.3 kPa)	[61]
	PVA/H_3_PO_4_	37.7 kPa^−1^	0.9977	350 kPa	23 ms	0.32 Pa	5000 (9.2 kPa)	[71d]
	PVA/H_3_PO_4_	1.2 kPa^−1^	0.999	150 kPa	2 ms	0.1 Pa	10 000 (20 kPa)	[71c]
	TPU/[EMIM][TFSA]	480.7 kPa^−1^	0.96	25kPa	10ms	‐	2000 (0.08N)	[75]
	PVA/H_3_PO_4_	519 kPa^−1^	‐	100 kPa	0.6ms	‐	10 000 (100 kPa)	[20c]
	P(VDF‐HFP)/[EMIM][TFSI]	1677.79 kPa^−1^		30 kPa	75 ms	50 Pa	10 000(1 kPa)	[44]
	P(VDF‐HFP)/[EMI][TFSA]	108.52 kPa^−1^	‐	275 kPa	55.8 ms	‐	5000(50 kPa)	[72b]
	PVA/[EMIM][OTF]/PU	66.3 nF kPa^−1^	‐	0.5 kPa	36 ms	‐	1000	[72a]
	PVA/H_3_PO_4_	810 kPa^−1^	‐	440kPa	2.74ms	‐	10 000 (200 kPa)	[28]
	PVA/H_3_PO_4_	365 kPa^−1^	0.981	1000kPa	30ms	1.7Pa	10 000 (20 kPa)	[38a]
	P([BMIM][SPA]‐co‐MA))‐HDDA	69.6 kPa^−1^	‐	1000kPa	6ms	‐	30 000 (300 kPa)	[50]
	P(AMT‐co‐MA)‐PMA	3.3 kPa^−1^	‐	1000kPa	3.8ms	‐	1000(400 kPa)	[88]
	P(DMAPS)‐Solketal	1100 kPa^−1^	‐	200kPa	20ms	‐	1000 (100 kPa)	[89]
	BA‐PEGMA/[EMIM][DCA]/PEGDA/TPO	14.1 kPa^−1^	‐	20kPa	20ms	‐	1000(14 kPa)	[20a]
	PEGDA/HEMA/[EMIM][OTF]	83.9 kPa^−1^	‐	100kPa	61ms	10Pa	5000 (1 kPa)	[39a]
	P(VDF‐HFP)/[EMIM][TFSI]	9.55 kPa^−1^	‐	8kPa	<52ms	<5Pa	700 (1.4 kPa)	[90]
	PVDF‐HFP/[EMIM][TFSI]/HMDA	4.5 kPa^−1^	‐	10kPa	50ms	0.2 Pa	500 (4 kPa)	[29]
	TPU/[EMIM][NTf_2_]	87.75 kPa^−1^	‐	170kPa	7.52ms	0.22Pa	1050 (0.1N)	[91]
	TPU/[EMIM][NTf_2_]	106.01 kPa^−1^	‐	77kPa	16ms	1.18Pa	2000 (0.5N)	[92]
	PUA/[VEIM]NTf_2_/TBC	40.39 nFkPa^−1^	‐	250kPa	34ms	0.59Pa	10 000	[93]
	P(AA‐AM)/MgCl_2_	0.06 kPa^−1^	‐	70kPa	320ms	26Pa	200(8 kPa)	[30]
	TPU/[EMIM][TFSI]	2.65 nF kPa^−1^	‐	10kPa	47ms	‐	200(0.5 kPa)	[55]
	P(VDF‐HFP)/PUA/[EMIM][TFSI]	Sn: 1.69 × 10^4^ pF kPa^−1^	‐	600kPa	Sn:38.7 ms	20 Pa	1000(−80 kPa)	[31]
		Sp: 2.52 × 10^3^ pF kPa^−1^	‐	−100kPa	Sp:0.9ms	0.05Pa	10 000(420 kPa)	
	PVA/H_3_PO_4_	Sn: 0.050 kPa⁻¹	‐	−92kPa	15ms	10Pa	4200(2.5 kPa)	[94]
		Sp: 0.156 kPa⁻¹		7kPa	‐	‐	‐	
	Water‐air interface/PBS solution	79.1 nF kPa^−1^	0.99646	15kPa	‐	‐	1000 (5 kPa)	[95]
	PVA/NaCl/Glycerol	58 kPa^−1^	‐	>100kPa	45ms	6.64Pa	2000(27 kPa)	[52]
	TPU/G4‐LiTFSI/[EMIM][TFSI]	47.16 kPa^−1^	‐	3.8MPa	41ms	9.8Pa	6000(1000 kPa)	[96]
	PVDF‐HFP/[EMIM][TFSI]	736.1 kPa^−1^	‐	300kPa	5.4ms	‐	5000(200 kPa)	[97]
	P(EA‐co‐AN)/[EMIM][TFSI]	2.48 kPa^−1^	0.998	2MPa	50ms	0.38Pa	2000(1 MPa)	[98]
	PVDF‐HFP/[BMIM][TFSI]	3.9 kPa^−1^	‐	1880kPa	‐	0.12Pa	500(40 kPa)	[99]
	TPU/[BMIM][TFSI]/PVDF‐HFP	185.09 kPa^−1^	0.9999	100 kPa	60ms	0.49Pa	1000(5 kPa)	[100]
Internal Porous Structure	PDMS/[EMIM][TFSI]	0.93 kPa^−1^	0.992	400 kPa	300 ms	‐	11 800 (12.5 kPa)	[21a]
	PU/[EMIM][TFSI]	5.28 nF kPa^−1^		118kPa	100ms	‐	‐	[54]
	PU/[BMIM]BF_4_	9280 kPa^−1^	‐	120kPa	10ms	‐	5000 (10 kPa)	[14]
	PEA‐*r*‐PS‐*r*‐PDVB/[EMIM][TFSI])	152.8 kPa^−1^	‐	400kPa		‐	6000 (10 kPa)	[21b]
	[EMIM][TCM]/PEGDA/cellulose fiber	20 nF N^−1^	‐	222.4N	‐	‐	‐	[76d]
	P(HEMA)/[EMIM][OTF]/paper fiber	1 nF kPa ^−1^cm^−2^	‐	200kPa	6ms	5.12Pa	5000 (10 Hz)	[76a]
	PVA/[EMIM][OTF]/paper	10 nF kPa ^−1^cm^−2^	0.996	25kPa	5ms	6.25Pa	1000(10 kPa)	[76b]
	TPU/[EMIM][TFSI]	221.9 kPa^−1^	‐	30kPa	300ms	2Pa	4000 (0.5 kPa)	[76c]
	CNFs/[BMIM]Cl	67.5 kPa^−1^	0.998	30kPa	84ms	0.5Pa	3000 (25 kPa)	[101]
	[BMIM]∙PF_6_/textile	4.46 kPa^−1^	‐	120kPa	39ms	‐	10 000 (1.5 kPa)	[53a]
	[BMIM]·PF_6_/Fabric	6.5 kPa^−1^	‐	175kPa	30ms	7.5Pa	5000 (50 kPa)	[53b]
	[EMIM][TFSI]/P(VDF‐HFP)/DMF)/fabric	31.4 kPa^−1^	‐	80kPa	45ms	140Pa	1500 (1 kPa)	[77]
	[EMIM][TFSI]/TPU	1 nF kPa^−1^	‐	150kPa	78ms	15Pa	2000 (10 kPa)	[40c]
	P(VDF‐HFP)/[EMIM][TFSI] nanofabric	114 nF kPa^−1^	‐	80mmHg	4.2ms	2.4Pa	‐	[13b]
	TPU/[EMIM][TFSI]	271.5 kPa^−1^	0.976	30kPa	30ms	<2Pa	4000 (10 kPa)	[40b]
	[BMIM][BF_4_]/TPU	8.14 kPa^−1^	‐	280 kPa	25ms	‐	1000(15% tensile rate)	[78c]
	TPU/[BMIM][BF_4_]	147.19 kPa^−1^	‐	85kPa	50ms	60Pa	10 000 (2 kPa)	[42]
	P(SBMA‐co‐AM)/Sepiolite/PEGDA	0.17 kPa^−1^	‐	140kPa	20ms	62Pa	2000 (2 kPa)	[78b]
	TPU/IL(EMIM][NTf_2_]	1.58 kPa^−1^	‐	120kPa	170ms	23Pa	6000 (25.5 kPa)	[46]
	SF/FA/Glycerol	138.5 kPa^−1^	‐	100kPa	17ms	1Pa	18 000 (2 kPa)	[47]
	[PBVI][TFSI]nanofibrous	0.49 kPa^−1^	‐		30ms	20Pa	300(0.5 Pa)	[78a]
	Rose petal	1.54 kPa^−1^	‐	115kPa	‐	0.6Pa	5000 (150 Pa)	[26]
	TPU/[EMIM][TFSI] ionogel fibers	13.3 kPa^−1^	‐	207kPa	9.9ms	1.16Pa	2000 (0.03N)	[39b]
	PU‐TEM/[EMIM][TFSI]	49 999.5 kPa^−1^	‐	360kPa	19ms	50Pa	30 000 (300 kPa)	[62]
	TSPU foam/EMImFSI	16.24 kPa^−1^	0.999	300kPa	18ms	0.44Pa	150 000 (100 kPa)	[102]
	PI/[PMlm][NTf_2_]	158.67 kPa^−1^	0.999	4000kPa	2.4ms	0.25Pa	150 000 (1500 kPa)	[103]
	P(VDF‐HFP)/DMF/EMIM][TFSI]	9.62 kPa^−1^	‐	500kPa	30ms	0.884Pa	1000(5 kPa)	[104]
	PEDOT:PSS/RSF/Bismuthene	2.31kPa^−1^	0.97	48kPa	60ms	0.45Pa	1000(120 kPa)	[105]
	PVDF‐HFP/[EMIM][TFSI]	49 kPa^−1^	0.999	1MPa	71.5ms	5Pa	10 000(1 MPa)	[106]
	TPU/[EMIM][TFSI]	5.067 kPa^−1^	‐	60kPa	28ms	‐	7000(8 kPa)	[107]
	PVA‐KOH	23.1 kPa^−1^	0.994	1000kPa	14.2ms	6.4Pa	12 000 (800 kPa)	[108]
	PU/PVDF/[BMIM][BF_4_]	422.22 kPa^−1^	‐	80kPa	150ms	‐	10 000 (10 kPa)	[109]
	PAM/AG/CaCl_2_	7.56 kPa^−1^	‐	200kPa	30ms	10Pa	5000 (60 kPa)	[48]
Multiscale Hierarchical Structure	PVA/H_3_PO_4_	2593 kPa ^−1^	0.983	3.36MPa	26ms	7.2 Pa	2700 (1 MPa)	[79a]
	PVA/H_3_PO_4_	13 786.2 kPa^−1^	‐	300 kPa	8ms	0.1 Pa	5000 (10 kPa)	[22a]
	[EMIM][TFSI]/wood	31.08 nF kPa^−1^	‐	500kPa	4ms	0.93Pa	10 000	[22b]
	PVDF‐HFP/[EMIM][TFSI]	8023.33 kPa^−1^	‐	320kPa	26ms	4.07Pa	8000 (4.1 kPa)	[79c]
	SA/PAM hydrogels	17 309 kPa^−1^	‐	372kPa	30ms	0.38Pa	5000 (300 kPa)	[79b]
	P(VDF‐HFP)/[EMIM][TFSI]	9.17 kPa^−1^	‐	2063 kPa	5ms	0.013Pa	5000(60 kPa)	[80]
	PDMA/PACMO	5182.8 kPa^−1^	‐	750 kPa	7ms	0.05Pa	2000 (200 kPa)	[82]
	PVA/H_3_PO_4_	49.1 kPa^−1^	0.995	485kPa	0.61ms	‐	5000 (300 kPa)	[25]
	PVA/[EMIM][OTF]	36 000 kPa^−1^	‐	300 kPa	40 ms	0.015 Pa	5000 (10 kPa)	[27]
	(PVDF‐HFP)/[EMIM][TFSI]	0.7291 kPa^−1^	0.9985	1000kPa	90ms	64Pa	8000 (60 kPa)	[110]
	NaCl‐PVA	25 548.24 kPa^−1^	0.99	127kPa	47.79ms	0.24Pa	10 000(7 kPa)	[111]
	PVA/[BMIM][BF_4_]	8.1 kPa^−1^	0.99	600kPa	20ms	2.1Pa	3000(26 kPa)	[81]

Note: Sn represents negative pressure sensing, Sp represents positive pressure sensing, “‐” means not mentioned in the references.

## 4 Superior Properties of Microstructure Interfacial Iontronic Pressure Sensors

Key parameters for evaluating the sensing performance of EDL pressure sensors include sensitivity, detection limit, linearity, working range, response speed, and stability. Building upon the structural innovations discussed in Section 3, such as microroughness surfaces, internal porous structures, and multiscale hierarchical designs, this section explores their impact on key sensing performance metrics.

### 4.1 High Sensitivity

Sensitivity is a key parameter in evaluating the accuracy and effectiveness of pressure sensors. For capacitive pressure sensors, sensitivity is mathematically defined as the relative change in capacitance (Δ*C*/*C*_0_) per unit pressure change (Δ*P*), represented as *S* = (△*C*/*C*_0_)/△*P*. This parameter determines the sensor's ability to detect subtle pressure variations, with higher sensitivity enabling finer detection.

**Figure 7:**
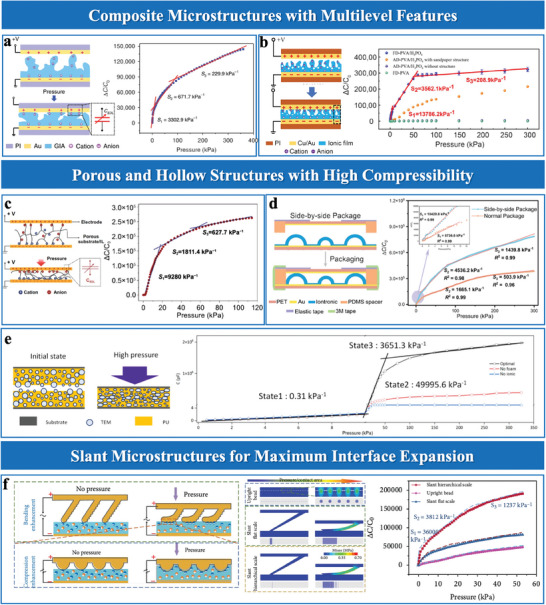
EDL Pressure sensors with high sensitivity: schematic diagram and sensing performance with various sensitive layers designs. a) Graded intra‐fillable microstructure. Reproduced with permission.^[^ ^70a^ ^]^ Copyright 2020, Springer Nature. b) Gradient microporous composite film. Reproduced with permission.^[^ ^22a^ ^]^ Copyright 2024, Elsevier. c) High‐porosity foam‐based structure. Reproduced with permission.^[^ ^14^ ^]^ Copyright 2021, Springer Nature. d) Graded hollow ball arch microstructure. Reproduced with permission.^[^ ^51b^ ^]^ Copyright 2024, Wiley‐VCH. e) Porosity structure using thermally expandable microspheres. Reproduced with permission.^[^ ^62^ ^]^ Copyright 2024, American Chemical Society. f) Schematic, simulation, and sensing performance of slant microstructure sensors. Reproduced with permission.^[^ ^27^ ^]^ Copyright 2022, Yongsong Luo et al.

In EDL pressure sensors, surface roughness significantly enhances sensitivity by increasing the effective contact area at the electrode‐dielectric interface. Under pressure, the contact area expands from an initial small area (*A*_0_) to a large area (*A*), leading to substantial capacitance changes (Δ*C*/*C*_0_). Micro‐structured surfaces, such as pyramids, pillars, and domes, are commonly fabricated using photolithography or mold‐assisted replication to enhance contact dynamics and capacitance response. Height variations in these microstructures amplify sensitivity by progressively expanding the contact area under applied pressure.^[^ ^51a^ ^]^ However, the intrinsic incompressibility of dielectric elastomers limits performance under higher pressures, requiring structural modifications to improve compressibility. Rationally designed microstructures address compressibility challenges by balancing contact area expansion and material flexibility. Bai et al.^[^ ^70a^ ^]^ introduced a graded intra‐fillable architecture that combined buckling microstructures with surface undercuts and grooves (**Figure** **7**a). Fabricated using a simple sandpaper‐molding technique, this innovative microstructure design effectively enhanced compressibility and sensitivity. This design achieved a sensitivity of 3302.9 kPa⁻¹ at low pressures and maintained a linear response with a sensitivity of 229.9 kPa⁻¹ under high pressures (>100 kPa). Building on the integration of various structural features. Li et al.^[^ ^22a^ ^]^ developed a gradient microporous film via freeze‐drying (Figure 7b), which featured a layered composite structure with a dense bottom layer, a porous upper layer, and a rough surface. This configuration enabled progressive contact area expansion and compressibility, achieving a sensitivity of 13 786.2 kPa^−1^ and a detection limit of 0.1 Pa.

Unlike rigid surface microstructures, pore or hollow structures exhibit enhanced compressibility due to reduced structural stiffness. Liu et al.^[^ ^14^ ^]^ utilized a gas foaming process to fabricate open‐cell PU foams with a porosity of 95.4% and a Young's modulus of 3.4 kPa. When infused with ionic liquid, this design achieved a sensitivity of 9280 kPa^−1^ (Figure 7c). The open‐cell design maintained compressibility under varying pressures, enhancing contact area and ion mobility. Inspired by alveoli biomechanics and architectural arch structures, Ding et al.^[^ ^51b^ ^]^ proposed a graded hollow ball arch microstructure (GHBA) using mold casting and moisture evaporation techniques (Figure 7d). This graded design, combined with side‐by‐side packaging, enhanced contact area expansion and pressure transfer, achieving a sensitivity of 10 420.8 kPa^−1^ over a pressure range of 0.2 Pa to 300 kPa. Building upon these concepts, Yang et al.^[^ ^62^ ^]^ introduced thermally expandable microspheres (TEM) as a method for creating dynamic porous structures. Fabricated via screen printing followed by thermal expansion at 120 °C, TEM created hollow microstructures that significantly increased the effective contact area and enhanced ion migration pathways. This design enabled high sensitivity of 49 999.5 kPa^−1^ and supported a broad detection range spanning 0–350 kPa (Figure 7e).

Most structural designs rely on axial compression deformation, which leads to structural hardening. This restricts the expansion of the electrode‐dielectric interface area and limits sensitivity. Inspired by the contact mechanics of gecko feet, Luo et al.^[^ ^27^ ^]^ engineered a slant hierarchical microstructure to maximize interface expansion under stress (Figure 7f). Fabricated using oblique lithography and transfer printing, the inclined microfeatures expanded the contact area dynamically during pressure application, mitigating pressure resistance. As a result, the sensor demonstrated a high sensitivity of 36 000 kPa⁻¹ over a broad detection range spanning 0 to 300 kPa.

In addition to microstructural innovations, incorporating pseudocapacitive materials effectively enhances sensitivity by leveraging their superior ion adsorption capabilities at the electrode‐electrolyte interface. Materials such as MXene,^[^ ^17^ ^]^ PEDOT,^[^ ^68^ ^]^ transition metal dichalcogenides (e.g., MOS_2_^[^ ^43^ ^]^), and graphene^[^ ^66^ ^]^ exhibit high capacitance variation, making them ideal for iontronic pressure sensors. For instance, an iontronic sensor with MnOx/MoS_2_ composite electrodes demonstrated exceptional sensitivity (58 853 kPa^−1^), primarily attributed to faradaic reactions that amplified capacitance changes, enabling precise detection of minute pressure variations.^[^ ^43^ ^]^

Integrating these strategies such as multilevel gradient microstructures, compressible porous/hollow structures, hierarchical microstructures maximizing interface expansion, and pseudocapacitive properties significantly improves the sensitivity of EDL pressure sensors. Advanced fabrication techniques, such as freeze‐drying and thermal expansion, have played a critical role in realizing these designs. These innovations address limitations in existing designs and enable highly responsive and accurate pressure sensing applications across diverse fields.

### 4.2 Limit of Detection

The limit of detection (LOD) represents the smallest pressure variation that causes a measurable capacitance change, typically defined by a specific signal‐to‐noise ratio. Unlike sensitivity, which quantifies the capacitance change per unit pressure, LOD emphasizes the ability to detect minimal forces, critical for applications like pulse wave monitoring and precise tactile sensing.

A key advantage of EDL‐based pressure sensors in achieving ultra‐low LOD lies in their utilization of high‐capacitance ionic materials, such as ionic gels, ionic liquids, and polymer electrolytes (e.g., PVA/H₃PO₄). These materials form ultra‐thin electric double layers (1–2 nm) at the electrode interface, enabling a highly responsive capacitance change under minimal deformations. This unique property makes EDL sensors superior to traditional capacitive sensors for detecting ultra‐low forces.

**Figure 8:**
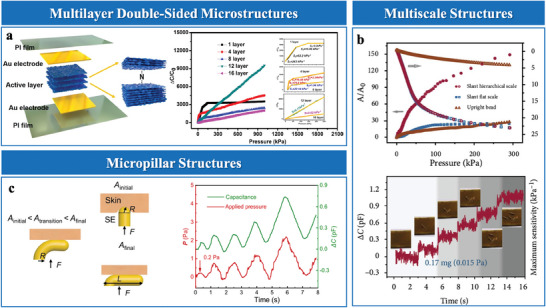
EDL Pressure sensors with low limit of detection. a) Layer‐dependent capacitance response of multilayer double‐sided microstructures under varying pressure. Reproduced with permission.^[^ ^80^ ^]^ Copyright 2021, American Chemical Society. b) Pressure‐responsive capacitance of gecko‐inspired multiscale structures. Reproduced with permission.^[^ ^27^ ^]^ Copyright 2022, Yongsong Luo et al. c) Buckling‐induced capacitance enhancement in micropillar arrays at minimal pressure levels. Reproduced with permission.^[^ ^59^ ^]^ Copyright 2021, Springer Nature.

Leveraging the benefits of ionic materials, advanced microstructural designs further minimize LOD by optimizing contact area and stress distribution. Hierarchical structures, such as multi‐layered or dual‐sided configurations, amplify capacitance through progressive contact area increases. Dual‐sided structures engage micro‐structured surfaces on both layers, enhancing sensitivity to minimal pressures via independent surface interactions. Multi‐layered structures, on the other hand, introduce incremental compression and stress distribution across stacked layers. By combining these features, a multilayer double‐sided micro‐structured iontronic film demonstrated an LOD of 0.013 Pa, with stable and cumulative capacitance changes under ultra‐low pressures (**Figure** **8**a).^[^ ^80^ ^]^

Multiscale structures integrate nano‐ and microscale features to enable a graded capacitance response to small pressures. In such architectures, nanoscale features initiate the initial capacitance change, while microscale features sustain and enhance the response as pressure increases. A gecko‐inspired structure, featuring inclined micro‐platelets and nanobeads, achieved an LOD of 0.015 Pa (Figure 8b).^[^ ^27^ ^]^ The inclined microplatelets bend under minimal pressure, dynamically expanding the contact area, while the nanobeads provide finer contact points for additional capacitance tuning.

Micropillar structures offer another approach for achieving low LOD, utilizing high‐aspect‐ratio designs that progressively buckle under slight forces. This behavior maximizes deformation and contact area, leading to enhanced capacitance. For instance, a micropillar‐based structure with a conductive surface coating achieved an LOD of 0.2 Pa (Figure 8c).^[^ ^59^ ^]^ Although less sensitive than those of hierarchical and multiscale designs, micropillar structures are advantageous for applications requiring simple mechanics and structural robustness, such as durable wearables.

In addition to microstructural design, additional enhancements, such as operating near an Exceptional Point (EP), are explored to further amplify detection capabilities. For example, an intracranial pressure sensor combining EDL technology with EP‐based enhancements achieved an LOD of 0.4 Pa, demonstrating its suitability for precise medical monitoring.^[^ ^112^ ^]^

### 4.3 Ultrabroad Linear Pressure Sensing Range

Incorporating microstructures into the dielectric or electrodes significantly enhances the sensitivity of EDL pressure sensors by increasing the contact area. However, this often leads to non‐linear capacitance‐pressure responses, especially at higher pressures where structural compressibility becomes a limiting factor.^[^ ^14^, ^70^, ^79^ ^]^ High linearity is crucial for simplifying data processing, circuit design, and improving response speed,^[^ ^113^ ^]^ key requirements for applications like robotic manipulation, aerodynamic pressure detection, and wearable plantar pressure monitoring.^[^ ^114^ ^]^

**Figure 9:**
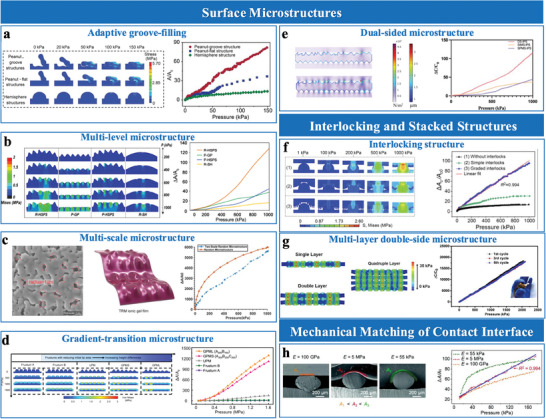
EDL Pressure sensors with ultrabroad linear sensing range. a) Adaptive groove‐filling structure. Reproduced with permission.^[^ ^71c^ ^]^ Copyright 2023, Wiley‐VCH. b) Multi‐level random microstructure. Reproduced with permission.^[^ ^79a^ ^]^ Copyright 2022, American Chemical Society. c) Multiscale microstructure. Reproduced with permission.^[^ ^40a^ ^]^ Copyright 2022, American Chemical Society. d) Gradient pyramidal microstructure. Reproduced with permission.^[^ ^20b^ ^]^ Copyright 2023, Springer Nature. e) Dual‐sided microstructure. Reproduced with permission.^[^ ^110^ ^]^ Copyright 2024, Elsevier. f) Interlocking structure. Reproduced with permission.^[^ ^25^ ^]^ Copyright 2022, American Chemical Society. g) Stacked structure. Reproduced with permission.^[^ ^80^ ^]^ Copyright 2021, American Chemical Society. h) Micro‐pillared structure. Reproduced with permission.^[^ ^70c^ ^]^ Copyright 2023, Springer Nature.

To mitigate the hardening effect at high pressures, intra‐filling microstructures, such as holes and grooves, are often employed to improve compressibility and linearity.^[^ ^38^, ^70^ ^]^ However, nonlinearity persists in such designs at higher pressures. Huang et al.^[^ ^71c^ ^]^ reported adaptive peanut‐groove microstructures using high‐precision 3D printing, enhancing linearity across a wide pressure range (R^2^ = 0.999, 0–150 kPa) (**Figure** **9**a). These structures redirect buckling forces into grooves, reducing structural resistance and accommodating deformation pathways under applied pressure. Conversely, groove‐free structures demonstrate reduced compressibility, resulting in a nonlinear contact area‐pressure relationship.

To improve linearity over broader pressure ranges, multi‐level and multiscale microstructures have been developed.^[^ ^40^, ^79^ ^]^ Chen et al.^[^ ^79a^ ^]^ engineered a hierarchical spinous structure (Figure 9b), featuring larger curved protrusions at the base to withstand high pressures, and smaller spinous protrusions at the top to maximize contact area changes, thereby enhancing both sensitivity and operational range. Wu et al.^[^ ^40a^ ^]^ developed a two‐scale random microstructure ionic gel film that integrates first‐scale micropores with a rough second‐scale surface (Figure 9c). This configuration enhances buffer capacity and uniform deformation, improving linearity compared to single‐scale structures.

Programmable gradient microstructures have recently been utilized for precise deformation control. Figure 9d illustrates CO_2_ laser‐fabricated gradient pyramidal microstructures, enabling controlled deformation under pressure. This design achieved a sensitivity of 33.7 kPa^−1^ and linearity (R^2^ = 0.99) across a broad sensing range up to 1.7 MPa. By ensuring uniform deformation within a single‐scale structure, this approach balances mechanical and electrical responses. Alternatively, Yuan et al.^[^ ^110^ ^]^ developed a double‐sided iontronic pressure sensor integrating irregular and pyramid microstructures on both sides of a PVDF‐HFP/EMIM active layer (Figure 9e). This design balances compression and mechanical alignment at the gel interface, achieving high linearity across pressure ranges of 100–760 kPa (R^2^ = 0.9975) and 760–1000 kPa (R^2^ = 0.9985).

Interlocking and stacked structures are effective strategies for improving linearity in EDL pressure sensors. Bai et al.^[^ ^25^ ^]^ reported a graded interlocking design featuring hemispherical arrays integrated with fine pillars within the ionic layer (Figure 9f). This configuration improves compressibility and ensures uniform stress distribution under varying pressures. Under increasing pressure, the micropillars gradually deform to fill gaps between the dielectric layer and electrode, mitigating structural stiffening and signal saturation. This design demonstrated a sensitivity of 49.1 kPa^−1^ and exhibited a high degree of linearity (R^2^ ≈0.998) over an extensive working range reaching 485 kPa. Moreover, Xiao et al.^[^ ^80^ ^]^ proposed a multilayer sensor incorporating double‐sided micro‐structured iontronic films as the dielectric layer (Figure 9g). This architecture, with randomly protruding microstructures between adjacent films, effectively distributes pressure loads while enhancing compressibility. This design enables an ultrabroad linear range exceeding 2.0 MPa, the highest‐pressure range being reported so far, with optimal linearity achieved when using 12 layers of iontronic films.

Finally, mechanical modulus matching at the electrode‐gel interface is crucial for maintaining high linearity. Lu et al.^[^ ^70c^ ^]^ reported an iontronic sensor featuring a micro‐pillared electrode paired with a soft ionic gel layer (Figure 9h), exhibiting high linearity (R^2^ ≈0.999) and sensitivity (33.16 kPa^−1^) across a pressure range of 12–176 kPa. The micropillars exhibit three deformation phases under pressure: initial contact at low pressures, buckling at moderate pressures, and post‐buckling at higher pressures. The key lies in matching Young's modulus of the electrode and gel, which allows subtle changes in the interfacial contact area to compensate for microstructural stiffening, ensuring consistent performance across a wide range of pressures.

However, achieving better linearity in EDL pressure sensors often comes at the cost of reduced sensitivity. This trade‐off fundamentally arises from the intrinsic mechanical properties and deformation behavior of the microstructures. To enhance linearity, microstructures are designed to deform uniformly across the pressure range, minimizing sharp changes in contact area. Although uniform deformation improves signal consistency, it diminishes the sharp, localized deformations crucial for high sensitivity, especially at lower pressures. Consequently, sensors optimized for high linearity often struggle to detect subtle pressure variations. Balancing this trade‐off is crucial for optimizing performance across a broad sensing range.

### 4.4 Stable Sensing Performance

Stability is critical for the reliable operation of EDL pressure sensors under complex mechanical and environmental conditions. However, achieving long‐term stability remains a multifaceted challenge, requiring interface robustness, resistance to creep and fatigue, and environmental resilience. These challenges are interconnected, for instance, mechanical failure at the electrode‐dielectric interface disrupts ion transport and exacerbates signal drift.^[^ ^103^ ^]^ Environmental factors, such as humidity fluctuations, extreme temperatures, and ion migration, accelerate interface degradation and compromise sensor performance.^[^ ^89^ ^]^ Addressing these interrelated challenges requires advanced material design, precise interface engineering, and environmental adaptability to ensure long‐term stability and reliability.

**Figure 10:**
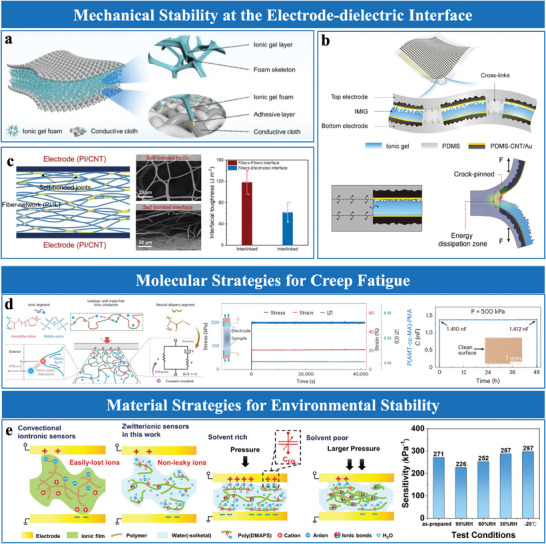
EDL pressure sensors with stable sensing performance. a) Schematic of the structure of the integrated sensor. Reproduced with permission.^[^ ^102^ ^]^ Copyright 2024, Wiley‐VCH. b) Integrated sensor design with two electrode layers and a PDMS interlayer embedded with IMIGs, featuring energy dissipation zones. Reproduced with permission.^[^ ^28^ ^]^ Copyright 2023, The American Association for the Advancement of Science. c) Schematic of functional layers, SEM images showing self‐bonded fiber‐fiber joints and fiber‐electrode interfaces, and the corresponding interface toughness. Reproduced with permission.^[^ ^103^ ^]^ Copyright 2024, American Chemical Society. d) Leakage‐ and creep‐free polyelectrolyte elastomer, variations in strain, impedance, and capacitance over time. Reproduced with permission.^[^ ^88^ ^]^ Copyright 2024, Springer Nature. e) Comparison of conventional iontronic sensors and zwitterionic hydrogel‐based sensors, demonstrating ion leakage prevention and stable performance under varying environmental conditions. Reproduced with permission.^[^ ^89^ ^]^ Copyright 2023, Wiley‐VCH.

Among these factors, maintaining mechanical stability at the electrode‐dielectric interface is critical for reliable sensor performance. Failures such as microstructure relaxation, delamination, or fatigue, often lead to stress concentrations, weakening mechanical integrity under repeated shear, compression, and bending stresses. To address these concerns, strategies like material integration,^[^ ^102^ ^]^ microstructure optimization,^[^ ^28^ ^]^ and unified material systems,^[^ ^103^ ^]^ have been proposed to tackle specific mechanical stability challenges. Material integration minimizes modulus mismatches and enhances interfacial bonding, serving as a foundational approach to improve mechanical stability. Zhu et al.^[^ ^102^ ^]^ developed an electromechanical integration strategy that combined thermosetting polyurethane (TSPU) foam with in situ ion‐gel polymerization (**Figure** **10**a). This method formed a cohesive multilayer interface, reducing modulus mismatches and stress concentrations. The integration of a TPU‐based adhesive layer further strengthened interfacial bonding, preventing delamination under cyclic loading and achieving high fatigue resistance over 150 000 cycles. However, localized stress concentrations may persist despite global modulus matching. To address these localized challenges, Shi et al.^[^ ^28^ ^]^ introduced microstructure optimization by designing perforated elastomeric matrices embedded with cross‐linked ionic gels. These structures dissipate energy and mitigate crack propagation through interhole wall deformation, ensuring stable performance over 10 000 cycles (Figure 10b). Building upon these approaches, unified material systems eliminate the need for adhesive layers and achieve intrinsically compatible interfaces. For instance, Li et al.^[^ ^103^ ^]^ proposed a unified design featuring self‐bonded porous ionic fiber structures with intrinsic material compatibility (Figure 10c). This architecture ensures uniform stress distribution, eliminating localized stress concentrations and delamination often seen in conventional layered designs. It withstands over 150 000 cycles under pressures up to 1500 kPa.

Beyond these strategies, advanced approaches like reversible metal coordination, chain entanglement, and biomimetic designs offer promising solutions for enhancing interfacial stability in EDL pressure sensors.^[^ ^115^ ^]^ Reversible metal‐ligand bonds at the electrode‐dielectric interface provide self‐healing capabilities and adaptive stress responses. These bonds can effectively address microcrack formation during cyclic loading. Polymer chain interpenetration within dielectric layers or at their interfaces may enhance toughness and mitigate crack propagation while maintaining flexibility, enhancing durability under repeated mechanical stress. Additionally, biomimetic microstructures inspired by designs like octopus suckers, can be applied to electrode or dielectric surfaces to distribute stress uniformly and increase adhesion under shear and bending stresses. While these strategies hold potential, further studies are required to assess their material compatibility, as well as their long‐term impact on ionic transport and overall sensor performance.

While mechanical stability addresses immediate interface challenges, long‐term performance degradation often stems from viscoelastic creep and material fatigue, both contributing to signal drift and structural instability.^[^ ^116^ ^]^ Molecular engineering offers targeted and effective solutions to address these challenges. He et al.^[^ ^88^ ^]^ developed a creep‐resistant polyelectrolyte elastomer with a tailored polymer network (Figure 10d). The material incorporates fixed cation segments to prevent ion migration and highly crosslinked neutral segments to suppress viscoelastic deformation. This design achieved stable sensing performance under static stress for 48 hours, with significantly reduced drift rates. Complementing this intrinsic approach, Yuan et al.^[^ ^50^ ^]^ introduced a polyelectrolyte elastomer system that combines robust material properties with microstructural enhancements. By integrating micro‐pyramid arrays, their approach redistributed stress and mitigated fatigue, enabling stable performance over 30 000 cycles at dynamic pressures of 300 kPa. Additional strategies such as dynamic crosslinking networks and nanofiller reinforcement also show promise in improving long‐term stability. Dynamic crosslinking networks adapt to mechanical deformation through reversible bonds, suppressing creep and mitigating fatigue under cyclic loading. Similarly, nanofiller reinforcement, incorporating materials like silica into polymer matrices, enhances mechanical strength and delays fatigue failure by restricting polymer chain mobility.

Environmental stability is equally critical for preserving the electrode‐dielectric interface and ensuring consistent performance of the sensors under fluctuating humidity and extreme temperatures. Effective strategies must address water loss, ion leakage, and interface degradation to maintain long‐term stability. Zhao et al.^[^ ^89^ ^]^ engineered zwitterionic hydrogels with electrostatic interactions for ion stabilization and hydrogen bonding for water retention (Figure 1). This design achieved stable performance across a wide humidity range of 30–90% RH and temperatures spanning −20–40 °C. These hydrogels also exhibited anti‐freezing properties, ensuring mechanical stability and ionic transport under subzero conditions. Similarly, He et al.^[^ ^20a^ ^]^ designed ionogels with a bicontinuous nanoscale network, facilitating efficient ionic pathways and enhancing mechanical resilience. These ionogels maintained stability under extreme conditions (−72 to 250 °C, 80% RH) and supported complex architectures via 3D printing. Emerging strategies expand these capabilities by employing stimuli‐responsive polymers that adapt dynamically to environmental changes, and biomimetic designs leveraging microstructural suction mechanisms for improved adhesion and stress distribution under high humidity or mechanical stress. Additionally, dynamic crosslinking networks with reversible bonds enable self‐healing and stress relaxation, effectively mitigating fatigue and creep under fluctuating conditions.

In summary, achieving stable sensing performance in EDL pressure sensors requires an integrated approach combining mechanical stability, resistance to creep and fatigue, and environmental resilience, particularly under varying humidity and temperature conditions. Material integration, microstructure optimization, and unified material systems address interface robustness, while molecular engineering mitigates long‐term deformation and fatigue. Furthermore, advanced materials like zwitterionic hydrogels and ionogels ensure durability under extreme environmental conditions by providing stable ionic transport and mechanical integrity. Together, these strategies highlight the critical role of material innovation and interface engineering in enhancing sensor reliability and longevity.

### 4.5 Fast Response and Low Hysteresis

**Figure 11:**
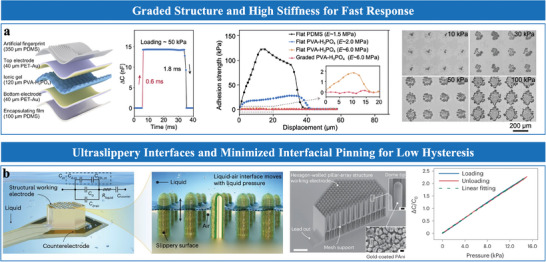
EDL pressure sensors with fast response and low hysteresis. a) Sensor architecture, response‐relaxation time, and adhesion strength influenced by the modulus of ionic gels and their contact behavior under various pressures. Reproduced with permission.^[^ ^20c^ ^]^ Copyright 2023, Springer Nature. b) Sensor design, SEM of a 3D‐printed hexagon‐walled pillar‐array device, and linear loading‐unloading performance. Reproduced with permission.^[^ ^95^ ^]^ Copyright 2023, Springer Nature.

Response speed is a key parameter in evaluating the dynamic performance and real‐time applicability of pressure sensors. The intrinsic properties of EDL, such as rapid polarization of the ionic film, provide a foundation for fast electrical response, enabling real‐time pressure detection.^[^ ^117^ ^]^ For EDL pressure sensors, response speed can be further improved through strategic microstructural design and material optimization. Minimizing the initial contact area at the interface reduces interfacial adhesion energy, promoting faster energy release and improving response speed. Selecting materials with suitable modulus, rapid deformation ability, and low viscoelasticity is also beneficial for weakening interfacial adhesion. For example, Bai et al.^[^ ^20c^ ^]^ demonstrated that response speed can be significantly enhanced by precisely controlling the stiffness of the ion gel (**Figure** **11**a). The gel's high elastic modulus (E = 5.5 MPa), combined with a graded microstructure, significantly reduced interfacial adhesion. As a result, the sensor exhibited a negligible adhesion strength, nearly 0 kPa, enabling rapid response to both static and dynamic stimuli across a wide frequency range (0–400 Hz).

In addition to response speed, the zero‐threshold effect and low hysteresis are critical for high performance dynamic sensing. The zero‐threshold characteristic allows sensors to detect even the smallest pressure changes without requiring an initial activation force, while low hysteresis ensures consistent and accurate responses over repeated loading and unloading cycles. Cheng et al.^[^ ^95^ ^]^ developed an aero‐elastic capacitive pressure sensor by leveraging a multiphasic solid‐liquid‐gas interface combined with advanced microstructural engineering (Figure 11b). The sensor's hexagon‐walled pillar‐array structure precisely modulated the liquid‐electrode contact area, enabling immediate responses to minimal pressure changes and eliminating the traditional pressure threshold. Additionally, the ultra‐slippery surface treatment significantly reduced interfacial pinning and contact angle hysteresis, achieving an exceptionally low hysteresis of 1.34 ± 0.20% and a high linearity (R^2^ = 0.99944). This design enhanced the sensor's dynamic response and ensured consistent performance in complex liquid environments.

**Table** **2** summarizes the common strategies to achieve EDL pressure sensors with excellent performance, such as sensitivity, detection limit, linear range, stability, response, and hysteresis. These performance criteria are inherently interdependent, and improving one parameter often comes at the expense of another. This presents a fundamental challenge in sensor design, necessitating strategic approaches to optimize and balance these trade‐offs. For example, microstructural designs that enhance sensitivity by increasing contact area may reduce the sensor's linear range at higher pressures due to localized stress saturation. To address this, advanced designs such as multilevel and graded structures are employed to balance sensitivity with a broader working range. Similarly, while robust interface designs improve mechanical stability, they may increase interfacial adhesion energy, leading to slower response speeds and higher hysteresis. Therefore, the use of materials with tailored moduli and low viscoelasticity, alongside precise microstructural control, is crucial for achieving rapid response and low hysteresis without compromising long‐term stability. Achieving optimal performance in EDL sensors requires a nuanced integration of materials and structural strategies, carefully balancing trade‐offs to ensure that the sensors can meet the demands of diverse applications.

**Table 2:** Microstructure and material designs and their impacts on the key sensing performance.

Performance	Structural/Material Strategy	Materials Property	Manufacturing	Limitations	Ref.
Sensitivity	Structures with height variation	Enhanced capacitance; dynamic contact area expansion	3D printing; sandpaper replication; lithography	Limited high‐pressure response;high cost	[34, 38, 41, 51, 87]
	Graded infillable structures	Enhanced compressibility	Sandpaper replication	Low durability under cyclic loading	[70a]
	Porous/hollow structures	Lightweight; enhanced compressibility and surface area	Thermal expansion; freeze‐drying; dual‐sided mold casting	Reduced durability; humidity sensitivity; scalability challenges	[22, 51, 62]
	Slant hierarchical structures	Dynamic contact area expansion	Oblique lithography; transfer printing	Deformation instability; complex fabrication	[27]
	Pseudocapacitive materials	High specific surface area; efficient charge transport	Solution‐based processes (e.g., spin coating, wet transfer); layer delamination	Environmental instability; mechanical fragility	[17, 43, 63]
LOD	Multilayer double‐sided structures	Dual‐sided activation;high compressibility	Layer stacking;dual‐sided imprinting	Mechanical complexity; durability under cyclic loading	[80]
	Slant hierarchical structures	Graded contact for minimal pressure detection	Oblique lithography; transfer printing	Precision alignment; scalability challenges	[27]
	High‐aspect‐ratio micropillars	Enhanced capacitance through progressive buckling	Replica molding with soft lithography	Pressure instability; fatigue under repetitive cycles	[59]
Wide linear range	Surface structures (multiscale, gradient, etc.)	Optimized stress distribution; enhanced compressibility	Laser ablation; mold casting; dual‐sided imprinting	Complex fabrication; mechanical instability; thickness constraints; high costs	[20, 40, 71, 79, 110]
	Interlocking/stacked structures	Uniform stress transmission; enhanced pressure distribution	Mold‐assisted stacking; 3D printing	Complex fabrication; increased thickness; durability challenges	[25, 80]
	Modulus‐matched interfaces	Stable interfacial deformation	Replica molding with soft lithography	Complex fabrication; limited material compatibility	[70c]
Stability	Self‐bonded reinforced interfaces	Enhanced toughness; crack resistance; uniform stress distribution	Imidization; molecular reinforcement integration; layered interface design	Limited scalability; complex fabrication	[28, 102, 103]
	Molecular engineering for creep resistance	Creep resistance; reduced signal drift	Copolymerization of ionic and neutral segments; physical reinforcement	Balancing mechanical robustness and ionic conductivity	[50, 88]
	Dynamic nano/molecular networks	High ionic conductivity; self‐healing; wide temperature range	Dynamic network construction via hydrogen bonding, ionic interactions, *etc*.	Balancing ionic conductivity and mechanical strength	[20, 58, 89]
Response and hysteresis	Graded structures with optimized modulus	Rapid energy recovery via low adhesion;	3D printing;replica molding	High precision; fabrication cost	[20, 25]
	Slippery multiphase interfaces	High wettability; low resistance	Nanocoatings with lubricants	Sensitive to humidity; complex processes	[95]

Note: The “Slant Hierarchical Structures” enhance both sensitivity and LOD

### 4.6 Other Characteristics

In addition to their core performance metrics, EDL pressure sensors exhibit additional unique characteristics, including high transparency,^[^ ^39^, ^49^, ^57^, ^118^ ^]^ comfortable wearability,^[^ ^19^, ^44^, ^47^, ^78^, ^96^ ^]^ selective sensing,^[^ ^20^, ^29^, ^37^, ^90^ ^]^ and multifunctional capabilities.^[^ ^19^, ^21^, ^42^, ^51^, ^91^, ^93^ ^]^ These features, achieved through advanced microengineering of the sensing layer, further enhance their versatility and expand their potential applications in emerging fields.

#### 4.6.1 High Transparency

**Figure 12:**
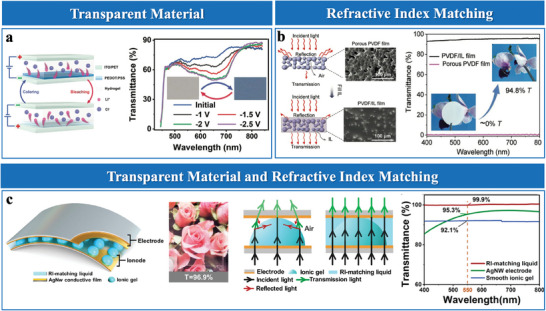
EDL pressure sensors with high transparency. a) Ionic migrations during coloring and bleaching processes, and transmission spectra at different coloring states. Reproduced with permission.^[^ ^57^ ^]^ Copyright 2023, Wiley‐VCH. b) Transition mechanism from opaque to transparent, and the corresponding visible spectra. Reproduced with permission.^[^ ^118^ ^]^ Copyright 2020, Wiley‐VCH. c) Device structure and light paths with and without RI‐mating liquid, and the associated visible spectra. Reproduced with permission.^[^ ^39a^ ^]^ Copyright 2022, Springer Nature.

High‐transparency flexible pressure sensors are essential for applications such as human–machine interfaces, smart windows, invisible robots, and touch screens. Iontronic sensors hold great promise in this field due to the inherent optical transparency of ionic functional materials. Nie et al.^[^ ^39a^ ^]^ pioneered a flexible transparent pressure sensor incorporating a transparent ionic gel with a transmittance of up to 99%. The gel covered the bottom electrode and was separated from the top electrode by a spacer layer. However, the optical absorption of the ITO electrode layers limited the overall light transmittance of the sensor to 77%. Similarly, Guo et al.^[^ ^57^ ^]^ developed a photocurable ionic hydrogel that combines iontronic pressure sensing and electrochromic properties (**Figure** **12**a). This design achieved high transparency, with transmittance ranging from 0% to 100% depending on the state of the electrochromic layer, and simplified the structure by allowing the hydrogel to function as both the dielectric and electrolyte layer. The developed device exhibited outstanding transmittance and pressure sensitivity, highlighting its potential as a candidate for next‐generation transparent pressure sensors.

The microstructural design of the interface significantly impacts the transparency of the device. Although surface microstructures can greatly enhance sensor sensitivity, they also induce strong light scattering, resulting in increased optical haze and reduced transparency of the dielectric layer.^[^ ^119^ ^]^ This effect primarily arises from the large refractive index difference between the matrix material and air at the rough interface.^[^ ^120^ ^]^ Replacing air in microstructured materials with refractive index‐matched fillers preserves interface continuity and minimizes light scattering.^[^ ^121^ ^]^ For example, Guo et al.^[^ ^118^ ^]^ developed a transparent iontronic material by impregnating a porous PVDF membrane with an ionic liquid matching its refractive index. This approach significantly increased transmittance from 0% to 94.8% (Figure 12b). However, optical losses at the interface between the ionic substances and sensing electrodes restricted the overall light transmittance of the device to 90.4%. Additionally, the improved optical properties of the PVDF structure reduced surface roughness, resulting in a lower sensitivity of 1.2 kPa^−1^. Tang et al.^[^ ^39a^ ^]^ further proposed an iontronic sensor by combining tunable surface structures with a refractive index matching strategy. This design achieved high sensitivity (83.9 kPa^−1^) and exceptional transparency (96.9%). The device incorporates an array of micro‐hemispherical transparent ionic gels sandwiched between transparent AgNW conductive films (Figure 12c). A non‐ionic refractive index matching liquid, introduced between the electrode and the dielectric layer, minimized light reflection at the interface, enhancing the sensor's transmittance.

#### 4.6.2 Wear Comfortability

For wearable devices, features like breathability, self‐cleaning, UV shielding, and antibacterial properties are essential for prolonged and comfortable use. Among these, comfort‐related properties such as breathability and flexibility are quantified by parameterslike ultra‐thin thickness (<100 µm), skin‐like flexibility (Young's modulus: 0.1–1 MPa), lightweight design (<10 mg cm^−2^), high air permeability (>1000 g m^−2^h^−1^), and strong hydrophobicity (water contact angle >120°).^[^ ^122^ ^]^ These parameters represent ideal design targets for wearable sensors to enhance skin compatibility by reducing irritation, maintaining stable contact, and managing sweat during prolonged use. However, many current devices still face challenges in achieving these metrics, particularly in terms of thickness, flexibility, and breathability.

**Figure 13:**
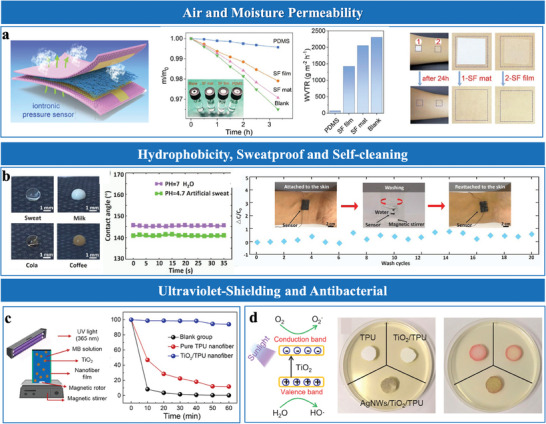
EDL pressure sensors with wear comfort. a) Nanofibrous sensor structure, water vapor transmission rates with sealed vials with various membranes, and photos showing SF mat on skin before and after 24 hours. Reproduced with permission.^[^ ^47^ ^]^ Copyright 2022, Elsevier. b) Hydrophobic sensor surface with liquid droplets photos, contact angle changes, and base capacitance stability after multiple washes. Reproduced with permission.^[^ ^44^ ^]^ Copyright 2023, Elsevier. c) UV shielding setup and decay curves of methylene blue absorption under different protections. Reproduced with permission.^[^ ^46^ ^]^ Copyright 2022, Elsevier. d) Schematic illustration of the antibacterial mechanism and bacterial growth observed on various nanofiber films, both prior to and 24 hours after incubation. Reproduced with permission.^[^ ^46^ ^]^ Copyright 2022, Elsevier.

Current devices often use sealing materials like Ecoflex, PDMS, TPU, PET, and PI, which hinder breathability and cause discomfort during extended wear.^[^ ^123^ ^]^ To address this, developing sensors with high breathability is essential. Wang et al.^[^ ^47^ ^]^ developed a breathable iontronic pressure sensor with high sensitivity, designed for continuous and long‐term blood pressure monitoring (**Figure** **13**a). The sensor employs an ionic fibrous SF/DES mat as the dielectric layer and incorporates micro‐structured SF/Au electrodes to enhance sensitivity. The SF/DES mat demonstrates a water vapor transmission rate (WVTR) of 2056 g m^−2^h^−1^, significantly higher than conventional materials like PDMS (71 g m^−2^h^−1^). Such high breathability prevents sweat accumulation at the skin‐sensor interface, thereby ensuring both signal stability and wear comfort during prolonged use. Additionally, Young's modulus of 8 MPa provided sufficient flexibility for conformal contact during use.

Hydrophobicity is equally critical to prevent sweat penetration and ensure stable operation in humid or underwater conditions. Sun et al.^[^ ^44^ ^]^ developed a tactile sensor with waterproof, breathable, and self‐cleaning properties, inspired by the lotus leaf structure (Figure 13b). The sensor incorporates an ion‐conductive gel electrolyte with through‐holes, sandwiched between two PDA/MXene/STA fabric electrodes, facilitating ionic transport and breathability. The STA micro‐sheets on the electrode surfaces form a rough micro‐nanostructure, which minimizes water adhesion. This structure achieves a water contact angle of 140.7°, enabling droplets to roll off easily, thus providing a self‐cleaning function. The sensor remains functional under artificial sweat and dynamic compression, demonstrating its applicability in high‐humidity and underwater environments.

Prolonged UV exposure during outdoor activities increases the risk of skin damage. Traditional wearable materials often lack UV protection and resistance to environmental pollutants. Cui et al.^[^ ^46^ ^]^ presented a breathable all‐nanofiber sensor using electrospun TPU nanofiber films. The outer layers, embedded with nano‐TiO₂, provide UV protection through the scattering and absorption of radiation (300‐420 nm) (Figure 13c). Nano‐TiO₂ also facilitates self‐cleaning via photocatalysis, decomposing organic pollutants like skin oils under UV light. Additionally, its combination with AgNWs imparts antibacterial properties, enhancing hygiene during wear. These features, combined with breathability and sensitivity, make it particularly suitable for outdoor activities and environments with high UV exposure.

In conclusion, the synergy of breathability, hydrophobicity, UV protection, and antibacterial properties establishes key targets for wearable sensors in challenging environments. While significant progress has been made in air permeability and hydrophobicity, further innovations in ultra‐thin and highly flexible materials are needed to fully meet wearability metrics.

#### 4.6.3 Directional and Selective Sensing

**Figure 14:**
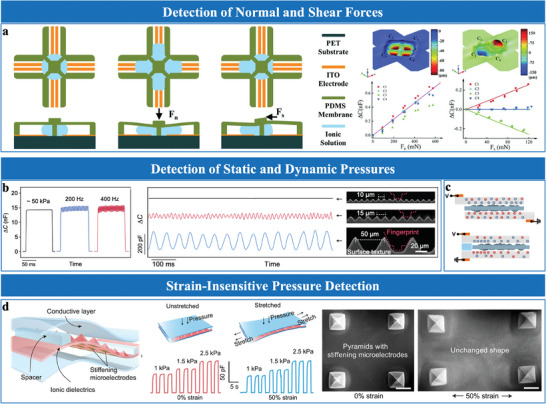
EDL pressure sensors with directional and selective sensing. a) Operation principle of a microfluidic tactile sensor under normal and shear forces, membrane deformation simulations, and sensitivity comparisons. Reproduced with permission.^[^ ^37^ ^]^ Copyright 2014, Royal Society of Chemistry. b) Detection of static pressure, vibrations at different frequencies, and signals from sliding on textured surfaces with different feature spacings. Reproduced with permission.^[^ ^20c^ ^]^ Copyright 2023, Springer Nature. c) Symmetrically and asymmetrically connected electrodes for directional sensing. Reproduced with permission.^[^ ^90^ ^]^ Copyright 2017, American Chemical Society. d) Sensor structure, operating states under unstretching and stretching, capacitance responses to repeated pressure, and SEM images of micropyramid structures at different strains. Reproduced with permission.^[^ ^29^ ^]^ Copyright 2021, The American Association for the Advancement of Science.

EDL pressure sensors have shown notable capabilities in detecting signals in specific directions, multi‐dimensional or omnidirectional configurations through innovative structural designs. For example, Pan et al.^[^ ^37^ ^]^ developed a tactile sensor utilizing microfluidic interfacial capacitive sensing for 3D force detection (**Figure** **14**a). The sensor integrates global and differential microfluidic sensing units with topologically engineered surfaces, enabling it to distinguish normal from shear forces. Under a normal load, the membrane deforms uniformly, leading to an equal increase in interfacial capacitance across all sensing elements. In contrast, a shear load causes differential deformation, leading to unequal capacitance changes among the sensing elements. These variations enable accurate detection of both the direction and magnitude of the applied mechanical load.

In addition to static pressure sensing, dynamic pressure detection is crucial for real‐time interactive applications, like robotics, virtual reality, and wearable health monitoring. Bai et al.^[^ ^20c^ ^]^ reported an iontronic slip‐sensor inspired by biological tactile perception (Figure 14b). The sensor incorporates a PDMS fingerprint, enabling simultaneous detection of static pressure up to 50 kPa and vibrations at 200 Hz (≈5 kPa) and 400 Hz superimposed on static pressure. The spatial resolution, determined by the ridge tip width of the artificial fingerprint (13 µm), enables detection of features with spacings as small as 15 µm. However, detecting finer features around 10 µm remains challenging due to structural resolution limitations in the fingerprint design. Similarly, Yoon et al.^[^ ^90^ ^]^ developed a highly sensitive piezocapacitive sensor capable of detecting both static and dynamic pressures through optimized electrode configurations. The sensor employs ion‐gel thin films combined with a CNT/PDMS composite dielectric layer. Symmetrical electrode configurations ensure consistent pressure sensitivity and uniform detection of both static and dynamic pressures across the sensor surface. In contrast, asymmetrical electrode configurations introduce an ion distribution gradient within the ion‐gel layers, creating a pressure‐sensitive gradient (Figure 14c). This allows the sensor to detect directional pressure changes and track lateral movements (e.g., sliding).

Stretchable pressure sensors are crucial for detecting interactions on soft and deformable surfaces, such as human skin, prostheses, or soft robots. However, most existing EDL pressure sensors suffer from accuracy deterioration when subjected to mechanical deformations like stretching or bending. These deformations cause variations in the contact area between the electrode and dielectric layer, disturbances in ionic pathways, and instabilities in the interfacial capacitance. These factors result in inaccuracies in pressure measurement. Su et al.^[^ ^29^ ^]^ tackled this issue by developing a strain‐insensitive EDL sensor with high pressure sensitivity and strain resilience (Figure 14d). The key innovation is the integration of rigid micro‐pyramid structures, which prevent deformation during stretching, preserving the sensor's geometric stability. Each pyramid's base is reinforced with high‐modulus stiffening electrodes to maintain the contact area between the electrodes and the dielectric layer during stretching. This design ensures capacitance stability. Additionally, flexible spacers are added to maintain consistent layer separation during deformation. These spacers act as mechanical buffers, further stabilizing capacitance under strain. Together, the rigid microstructures and flexible spacers decouple mechanical deformation from the sensing mechanism, ensuring reliable capacitance under normal pressure, even at varying strain levels.

#### 4.6.4 Multifunctional Sensing Capabilities

Recent advancements in intelligent technologies have enhanced the multifunctionality of EDL pressure sensors, enabling them to detect diverse stimuli, including pressure, temperature,^[^ ^42^ ^]^ proximity,^[^ ^124^ ^]^ and chemical signals.^[^ ^125^ ^]^ Such multifunctionality greatly broadens their applications in clinical diagnostics, environmental monitoring, and advanced human–machine interfaces.

Pressure and temperature are fundamental parameters in diverse applications. In the field of EDL pressure sensors, TPU/ILs ionic gels are widely employed as dielectric materials due to their temperature‐dependent dielectric properties.^[^ ^39^, ^91^, ^92^ ^]^ For example, Li et al.^[^ ^91^ ^]^ developed an iontronic sensor with a TPU/ILs dielectric layer, achieving dual‐mode pressure and temperature sensing. This design leverages the temperature‐dependent dielectric constant of the TPU/ILs layer to enhance temperature sensing, while interfacial contact area changes optimize pressure sensitivity. However, the shared reliance on capacitance for both parameters introduces challenges under simultaneous stimuli, necessitating signal decoupling strategies to ensure accurate multimodal detection.

**Figure 15:**
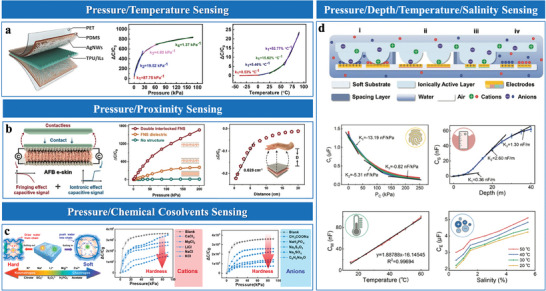
EDL pressure sensors with multifunctional sensing capabilities. a) Structural diagram and pressure/temperature sensing performance. Reproduced with permission.^[^ ^91^ ^]^ Copyright 2022, Elsevier. b) Test platform setup and capacitance responses in proximity and pressure modes. Reproduced with permission.^[^ ^19^ ^]^ Copyright 2023, Wiley‐VCH. c) Hoffmeister effect on hydrogel elasticity and capacitance‐pressure response curves in different salts. Reproduced with permission.^[^ ^51a^ ^]^ Copyright 2023, American Chemical Society. d) Device architecture of iontronic sensing units, with response curves for contact pressure, hydraulic pressure, temperature, and salinity. Reproduced with permission.^[^ ^93^ ^]^ Copyright 2022, Wiley‐VCH.

In addition to temperature sensing, proximity detection enriches the perceptual dimensions of EDL sensors.^[^ ^59^ ^]^ For example, the double interlocked structure in the all‐fabric bionic e‐skin developed by Niu et al.^[^ ^19^ ^]^ enhances both pressure and proximity sensing by improving stress distribution and expanding the effective contact area (**Figure** **15**b). This structural optimization increases pressure sensitivity and strengthens the fringing electric field, enabling precise proximity detection up to 20 cm. While this design integrates proximity sensing, its reliance on capacitive signals for both modalities introduces potential cross‐talk under simultaneous stimuli, necessitating advanced signal processing techniques to ensure reliable operation.

The detection of stress and chemical signals creates novel opportunities for applications in clinical diagnostics and sports monitoring. Li et al.^[^ ^51a^ ^]^ developed an iontronic sensor based on the Hofmeister effect, where hydrated ions modulate the rigidity of a conductive hydrogel, enabling the conversion of chemical signals into mechanical responses (Figure 15c). This innovative design exhibits promise for real‐time chemical and biological signal detection, offering applications in beverage classification and cosolvent analysis. However, the overlapping mechanical responses to stress and chemical stimuli underscore the critical need for materials with tailored selectivity or structurally optimized designs to decouple these responses and enhance multimodal sensing specificity.

Multimodal environmental sensing capabilities are essential for exploration and monitoring in aquatic environments.^[^ ^126^ ^]^ Cheng et al.^[^ ^93^ ^]^ proposed a multimodal sensor, referred to as aquatic skin, which integrates multiple iontronic sensing units (ISUs) to detect contact pressure, depth, temperature, and salinity within a single platform (Figure 15d). The device combines standard ISUs for depth detection and inverse ISUs for contact pressure and tactile mapping, leveraging hydraulic balance to minimize cross‐talk. Material and environmental ISUs contribute to temperature and salinity detection, respectively, with function‐specific layers. This compartmentalized design effectively isolates sensing modalities, reducing signal interference and enabling high‐resolution, multi‐parametric monitoring.

Despite progress in multimodal EDL sensors, the issue of overlapping signal outputs remains unresolved in most designs. Future research should focus on targeted approaches, such as selective materials, hybrid architectures, and advanced signal processing techniques, to ensure reliable multimodal sensing under simultaneous stimuli.

**Table** **3** summarizes key strategies for developing EDL pressure sensors with additional features, including high transparency, wearability, directional/selective sensing, and multifunctional sensing capabilities.

**Table 3:** Microstructural and material designs and their impacts on other characteristics.

Performance	Structural/Material Strategy	Materials Property	Manufacturing	Limitations	Ref.
Transparency	Transparent ionic materials	High clarity; low scattering	Solution casting	Transparency reduction by electrodes	[57]
	Refractive index matching	Minimal haze; smooth interfaces	Refractive index‐matching ionic liquid filling	Transparency loss due to unfilled pores and refractive mismatch	[118]
Wear comfort	Nanofiber structures	Breathability; moisture control	Electrospinning	Reduced signal transmission; environmental instability	[40, 47, 77]
	Micro/nano structures;low‐energy coatings;drainage channels	Waterproof; self‐cleaning	Solution spraying; dip coating	Layer integration challenges; durability issues	[44, 78]
	UV‐blockers; antibacterial coatings	UV protection; antibacterial	Solution coating; surface treatment	High cost; complex integration	[46]
Directional/selective sensing	Microfluidic interfaces for force differentiation	Sensitive for precise directional detection	Laser micromachining; photolithography, plasma bonding.	Mechanical instability; leakage risk	[37]
	Fingerprint‐like elastomer	Spatiotemporal resolution for slip and pressure	3D printing, demolding	Fabrication complexity	[20c]
	Asymmetrical electrode configurations	Directional sensitivity for force differentiation	Precise electrode placement	High alignment precision challenges	[90]
	Rigid elements and flexible spacers	Maintain contact geometry under strain	Rigid microstructure molding; precise spacer placement	Fabrication complexity; integration challenges; delamination risks	[29]
Multifunctional sensing	Temperature‐responsive ionic gels	Temperature‐pressure sensitivity	Solution processing	Material calibration required	[39, 91, 92]
	Fringe‐field structured iontronic interfaces	High dielectric constant; fringe‐field sensitivity for proximity‐pressure detection	Hydrothermal synthesis; template molding	Fringing field range limits	[19, 21, 124]
	Hydrogel‐based Hofmeister effect	Chemical signal detection	Hydrogel synthesis	Ionic species sensitivity limits	[51a]
	Partitioned iontronic structures	Comprehensive environmental sensing	Photolithography; polymer reflow;encapsulation	Fabrication complexity;potential interference	[93]

## 5 Microstructure Fabrication Strategies for Interfacial Iontronic Pressure Sensors

Selecting appropriate fabrication techniques is crucial for optimizing the performance of EDL‐based pressure sensors, as the resulting microstructure significantly influences sensitivity, detection limit, and other key properties. For example, precise microstructures fabricated via photolithography significantly enhance the effective electrode surface area, thereby boosting sensitivity, while gas bubble‐assisted methods are highly effective in creating porous structures that improve compressibility and detection limit. This section reviews progress in micropatterning and externally assisted fabrication strategies, emphasizing their impact on microstructure design and performance improvements.

### 5.1 Micropatterned Fabrication of Structured Sensing Materials

Micropatterning techniques, particularly photolithography and printing, are widely used to fabricate micro‐structured materials in EDL pressure sensors. These methods allow precise geometric control, directly influencing critical sensor properties, such as sensitivity, detection limit, and linear range. By tailoring the surface topography, micropatterning techniques optimize electrode‐electrolyte interactions, enhance stress distribution, and improve structural compressibility.

**Figure 16:**
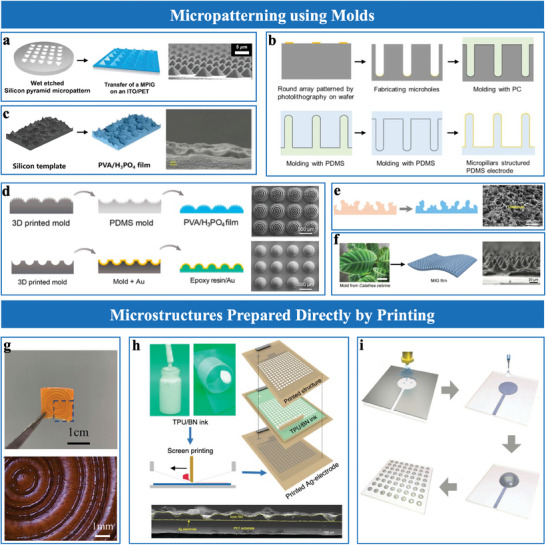
Micropatterned fabrication of structured sensing materials. a) Photolithography‐based preparation of micro‐pyramidal molds. Reproduced with permission.^[^ ^38b^ ^]^ Copyright 2017, American Chemical Society. b) Micropillar shape molds via photolithography. Reproduced with permission.^[^ ^70c^ ^]^ Copyright 2023, Springer Nature. c) Hierarchical arete architecture prepared using photolithography. Reproduced with permission.^[^ ^45^ ^]^ Copyright 2022, Wiley‐VCH. d) Sensitive structures fabricated with high‐precision 3D printing molds. Reproduced with permission.^[^ ^25^ ^]^ Copyright 2022, American Chemical Society. e) Sandpaper molding for micro‐structured surfaces. Reproduced with permission.^[^ ^70a^ ^]^ Copyright 2020, Springer Nature. f) Natural plants templates for structural replication. Reproduced with permission.^[^ ^61^ ^]^ Copyright 2018, WILEY‐VCH. g) 3D printed structural hydrogels. Reproduced with permission.^[^ ^30^ ^]^ Copyright 2021, IOP Publishing. h) Silver electrode and ionic film fabricated via screen printing. Reproduced with permission.^[^ ^16^ ^]^ Copyright 2023, Springer Nature. i) All‐printed fabrication process for an ionic mechanotransducer array. Reproduced with permission.^[^ ^55^ ^]^ Copyright 2020 WILEY‐VCH.

Photolithography is a widely adopted approach for creating micro‐structured molds, such as micro‐pyramids,^[^ ^29^, ^38^, ^49^, ^50^, ^63^ ^]^ micro‐pillars^[^ ^59^, ^70^ ^]^ and random microstructures.^[^ ^45^, ^87^ ^]^ These molds are typically used to shape sensing materials with precise geometries, maximizing surface area and improving the capacitive response for enhanced sensitivity. For instance, micro‐pyramidal structures, fabricated via photolithography and etching (**Figure** **16**a), provide increased surface area and dynamic contact adaptability, significantly improving sensitivity and detection limits.^[^ ^38b^ ^]^ Micropillar arrays, another widely investigated structure, demonstrate the precision and versatility of photolithography in fabricating high‐aspect‐ratio microstructures. These structures enhance electrode‐electrolyte interactions, contributing to improved sensitivity and detection limits in EDL pressure sensors. Lu et al.^[^ ^70c^ ^]^ employed photolithography and catalytic etching to fabricate a silicon‐based micro‐hole array with a height‐to‐radius aspect ratio of 10:1. This array was then molded into PDMS micro‐pillared structures, enhancing pressure sensitivity by optimizing the electrode‐electrolyte contact area (Figure 16b). Building on this approach, Li et al.^[^ ^91^ ^]^ fabricated dielectric layers with cup‐shaped microcolumns array. This was achieved by utilizing cylindrical grooves on a silicon wafer to guide the transition between the Cassie‐Baxter and Wenzel wetting states, thereby maximizing contact area under pressure. In addition to standard microstructures, hierarchical structures can be achieved through chemical etching, as demonstrated by the fabrication of silicon templates with arete architectures (Figure 16c)^[^ ^45^ ^]^ Beyond geometric versatility, advanced photolithographic techniques enables further customization. Luo et al.^[^ ^27^ ^]^ adjusted the incident angle of UV light to create photoresist templates with tilted microstructures. Cheng et al.^[^ ^93^ ^]^ utilized thermal reflow to transform cylindrical patterns into hemispheric or plateau structures, achieving smoother edges and uniform surface profiles.

In addition to photolithography, high‐precision 3D printing presents a promising alternative for fabricating complex, tailored microstructures. Techniques such as micro‐stereolithography enable the creation of graded or hierarchical structures, which are beneficial for extending linear sensing ranges. For example, Bai et al.^[^ ^25^ ^]^ fabricated a hemispheric graded structure using 3D printing (Figure 16d), which enhanced the sensor's performance across a wide pressure range. Unlike photolithography, 3D printing supports multi‐material and complex geometries without requiring intricate templates, suiting structurally adaptive applications. However, the resolution limitations of current 3D printing technologies constrain its application in submicron‐scale features. Advancements in nanoscale resolution and multi‐material printing are expected to address these limitations, enabling the integration of functional materials with microstructures for improved performance.

Low‐cost template‐based methods, such as sandpaper molding and natural structure replication, provide cost‐effective and scalable alternatives for micropatterning. These methods utilize naturally derived or readily accessible templates to fabricate microstructures that are both simple and functional. For instance, graded infillable architectures fabricated using sandpaper templates demonstrate enhanced compressibility and structural stability under load (Figure 16e).^[^ ^70a^ ^]^ Similarly, micro‐cone structures replicated from plant leaves have been shown to optimize sensitivity for low‐pressure sensing applications (Figure 16f).^[^ ^61^ ^]^ While these methods lack the precision of photolithography or 3D printing, they are highly adaptable and provide cost‐effective solutions for applications requiring moderate performance improvements.

Printing‐based approaches, such as digital light processing (DLP) and screen‐printing, have broadened micropatterning capabilities by enabling the fabrication of patterned materials without the need for molds or templates. For example, fingerprint‐like micro‐structured hydrogel films printed via DLP served as sensing components in EDL pressure sensors (Figure 16g).^[^ ^30^ ^]^ Screen‐printing facilitates the fabrication of large‐area flexible pressure sensor arrays due to their scalability and adaptability. Yang et al.^[^ ^16^ ^]^ demonstrated this technique by incorporating hexagonal *h*‐BN into ionic ink, where *h*‐BN functioned as an ionic liquid reservoir and viscosity regulator, enabling the printing of high‐quality micro‐structured ionic films and electrodes on flexible substrates (Figure 16h). Similarly, Kim et al.^[^ ^55^ ^]^ developed an ionic mechanotransducer array with high aspect ratio dome structures, significantly enhancing pressure sensitivity by optimizing surface contact mechanics (Figure 16i). Further performance improvements were realized by optimizing printing parameters, such as resolution and layer thickness, and refining material properties, including viscosity and conductivity.

In summary, micropatterning techniques advance EDL pressure sensors by enabling customized microstructures for optimized performance. Photolithography offers high precision but limited scalability, 3D printing provides flexibility, and template‐based methods offer cost‐effective alternatives. Printing techniques enhance scalability, supporting large‐scale and diverse applications. Integrating these complementary approaches could lead to versatile, high‐performance materials for next‐generation sensors.

### 5.2 Externally Assisted Fabrication of Structured Sensing Materials

Externally assisted fabrication methods encompass mechanical force‐assisted, thermal‐induced, electric field‐assisted, gas bubble‐assisted, and template‐assisted approaches. These techniques enable precise control over material properties, facilitating the creation of microstructures with tailored characteristics.

**Figure 17:**
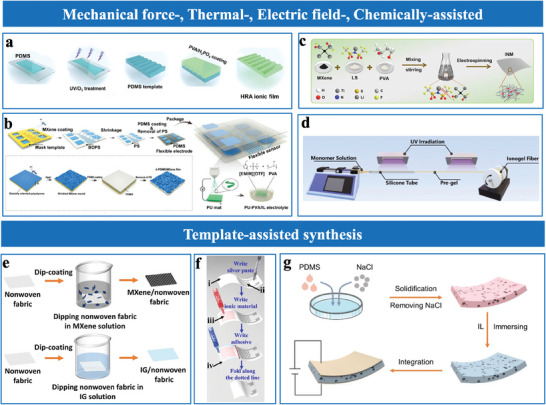
Externally assisted fabrication of structured sensing materials. a) Fabrication of hill‐ridge architecture film using a PDMS mold with pre‐stretching and release. Reproduced with permission.^[^ ^71d^ ^]^ Copyright 2021, Wiley‐VCH. b) Preparation of PDMS/MXene‐based touch sensors with hierarchical wrinkled MXene layer. Reproduced with permission.^[^ ^72a^ ^]^ Copyright 2021, American Chemical Society. c) Ionic nanofibrous membrane fabrication process. Reproduced with permission.^[^ ^56^ ^]^ Copyright 2021, American Chemical Society. d) Dynamic reactive spinning of ionogel fibers via two‐step photopolymerization and post‐drawing. Reproduced with permission.^[^ ^78b^ ^]^ Copyright 2023, Elsevier. e) Fabrication of MXene/nonwoven fabric electrode and ionogel/nonwoven fabric electrolyte. Reproduced with permission.^[^ ^77^ ^]^ Copyright 2022, American Chemical Society. f) Handwriting iontronic pressure sensing origami processing flow. Reproduced with permission.^[^ ^76a^ ^]^ Copyright 2019, American Chemical Society. g) Fabrication of porous PDMS active layer. Reproduced with permission.^[^ ^21a^ ^]^ Copyright 2023, Wiley‐VCH.

Mechanical force‐assisted fabrication, such as pre‐stretching and compressing, is effective for producing wrinkled or buckled microstructures.^[^ ^71^, ^79^ ^]^ For instance, Qin et al.^[^ ^71d^ ^]^ fabricated a hill‐ridge architecture (HRA)‐based iontronic sensor by pre‐stretching and releasing a PDMS film to form sinusoidal folds (**Figure** **17**a). These folds served as templates for the HRA iontronic film, improving mechanical flexibility and pressure sensitivity. However, excessive pre‐stretching reduced sensitivity, emphasizing the need to optimize strain levels for balanced performance and durability.

Thermal induction is also widely adopted for generating wrinkle patterns and hierarchical structures on flexible substrates, enabling microstructures that enhance mechanical flexibility and electrochemical efficiency. For example, Chen et al.^[^ ^72a^ ^]^ fabricated a PDMS/MXene composite with hierarchical wrinkles using thermal treatment, achieving high stretchability and strain‐invariant conductivity (Figure 17b). These hierarchical structures increased the effective ion‐electrode interface area and stabilized ionic pathways under deformation, significantly enhancing the composite's dynamic response and pressure sensitivity. However, achieving uniform and scalable wrinkle patterns remains challenging and might involve improvements in fabrication techniques, thermal management, or other strategies for large‐scale applications.

Electric field‐assisted fabrication, such as electrospinning,^[^ ^13^, ^42^, ^46^, ^56^, ^78^ ^]^ enables the production of polymer filaments with controlled diameters. These filaments form 2D membranes or 3D fabrics with high surface area and tunable porosity. For instance, electrospinning synthesized a hybrid ionic nanofibrous membrane by incorporating MXene and lithium sulfonamide salts into a PVA matrix (Figure 17c).^[^ ^56^ ^]^ The uniform dispersion of MXene enhanced ion confinement and mobility, improving the sensor's ionic conductivity and dynamic response. Moreover, chemically assisted spinning technologies, such as wet spinning,^[^ ^39b^ ^]^ and dynamic reactive spinning,^[^ ^78b^ ^]^ further expand the functionality and versatility of fiber‐based materials. Zhao et al.^[^ ^78b^ ^]^ developed a dynamic reactive spinning method to continuously fabricate tough ionogel fibers reinforced with orientated nanomaterials (Figure 17d), contributing to enhanced mechanical stability and consistent ion transport under deformation.

Gas bubble‐assisted fabrication offers a simple yet effective approach for creating porous structures with enhanced compressibility. Liu et al.^[^ ^14^ ^]^ fabricated open‐cell polyurethane (PU) foams with high porosity using this method. These foams were subsequently infused with ionic liquid to form the ionic layer. This design lowered the material's modulus (≈3.4 kPa), enhancing ion‐electrode interactions and achieving a high‐pressure resolution of 0.125%, making it particularly suited for low‐pressure sensing applications. However, achieving uniform pore distribution and scalable production remains challenging.

Template‐assisted fabrication includes template‐directed and sacrificial template approaches. Template‐directed fabrication involves depositing active materials (e.g., ionic materials) onto substrates such as textiles and paper via coating or dip‐coating processes.^[^ ^44^, ^53^, ^104^ ^]^ For example, Sun et al.^[^ ^77^ ^]^ presented a textile‐based supercapacitive pressure sensor using MXene‐decorated nonwoven fabrics as electrodes and ionic gel‐infiltrated nonwoven fabric as the electrolyte (Figure 17e). Similarly, modifying common paper with functional materials enables the fabrication of low‐cost, pressure‐sensitive devices.^[^ ^76a,d^ ^]^ Li et al.^[^ ^76a^ ^]^ introduced an iontronic origami pressure sensor, using handwriting to pattern ionic‐electrode interfaces, enabling flexible and efficient fabrication (Figure 17f). The self‐sacrificial template method uses dissolvable materials, like sugar or salt, to create porous or hollow structures.^[^ ^21^ ^]^ For instance, a flexible iontronic skin with a porous PDMS active layer was fabricated using salt as a sacrificial template to form interconnected pores (Figure 17g).^[^ ^21a^ ^]^ By varying the amount of salt, the porosity of the PDMS layer was adjusted from 64% to 90%, enhancing mechanical sensitivity and compressibility by increasing deformation capacity. The ionic liquid‐coated pore walls further improved ionic conductivity and capacitive response by enlarging the effective electrode‐ion interface area.

In summary, mechanical force‐assisted and thermal induction methods generate wrinkle patterns and hierarchical structures, enhancing flexibility and dynamic response. Electric field‐assisted techniques produce fibrous networks with high surface area and tunable porosity, while gas bubble‐assisted and template‐assisted approaches create porous structures to improve compressibility and ion‐electrode interactions. Despite these advancements, challenges such as scalability and reproducibility persist. Integrating these techniques could overcome these limitations, driving the development of scalable, high‐performance EDL pressure sensors.

### 5.3 Potential of Fabricating 3D Surface Sensors and Standalone Stretchable Platforms

The demand for pressure sensors on 3D curved surfaces is critical for applications in wearable electronics, robotic skins, and smart textiles, where sensors must conform to dynamic surfaces like human skin or robotic limbs. Traditional pressure sensors, designed primarily for flat substrates, struggle to adapt to complex geometries due to material uniformity, stability, and performance issues under deformation. In recent years, the fabrication of devices on curved surfaces has become increasingly prevalent, addressing issues like adhesion and material discontinuity. This approach allows sensors to conform more effectively to dynamic geometries while maintaining stable performance under deformation.

The fabrication of EDL pressure sensors on curved surfaces requires precise material selection and structural design. A critical aspect is reducing sensor thickness to enhance flexibility and improve conformity to 3D curved surfaces. While minimizing thickness enhances adaptability, maintaining a stable gap between the electrodes and the dielectric layer remains essential. An excessively small or unstable gap may lead to direct electrode‐dielectric contact, resulting in performance degradation. Dynamic surface deformation can further exacerbate this issue, causing inconsistent contact areas and inaccurate measurements. Achieving  precise electrode‐dielectric spacing requires high fabrication precision. Techniques like transfer printing and direct printing provide precise layer thickness and alignment control, ensuring consistent spacing even on dynamic surfaces. Additionally, using stretchable materials for the dielectric layer and electrodes, such as PDMS, PU, and conductive hydrogels, can effectively maintain stable spacing during deformation. This approach prevents layer contact and ensures reliable sensor performance under mechanical stress.

Furthermore, structural optimization is crucial for improving EDL sensors’ adaptability to dynamic surfaces. Geometrical designs, such as serpentine or mesh‐like structures, evenly distribute mechanical stress, improving flexibility and operational stability. Additionally, pre‐stretching and releasing techniques create conformal microstructures for small‐radius curved surfaces, improving adaptability to complex geometries.

Beyond individual sensor fabrication, the integration of standalone stretchable platforms is a growing focus in sensor technology. These platforms integrate sensors, circuits, and power management components into a single stretchable system, operating independently from external substrates. This approach is suited for self‐contained wearable devices and soft robotics, enabling multifunctional operation. Stretchable substrates made from PDMS and conductive hydrogels are commonly used to build these platforms, ensuring flexibility and robustness under deformation. Pre‐stretching and buckling techniques can be employed to preserve the functionality of the integrated sensors and circuits even on highly dynamic surfaces. The development of such platforms could transform applications in health monitoring, interactive textiles, and robotic systems by offering sensitivity, efficiency, and autonomy.

To ensure reliable sensor fixation, biomimetic adhesives inspired by systems like gecko feet or octopus suckers enable robust and reversible adhesion on dynamic surface. In humid or mechanically dynamic environments, hydrogel‐assisted fixation offers strong adhesion and flexibility, preventing delamination and ensuring consistent performance.

## 6 Sensor Comparison and Selection Guidelines

The performance of EDL pressure sensors is closely tied to their structural designs, which determine key parameters like sensitivity, detection limit, pressure range, response time, and durability. Each structural configuration offers  distinct advantages and limitations, shaping its suitability for specific applications. Understanding the trade‐offs among these parameters is critical to optimizing sensor selection for specific applications.

Nanoscale structures, characterized by high surface area and enhanced ion interactions, enable high sensitivity and low detection limits. These features make them suitable for subtle pressure sensing in healthcare applications, such as pulse wave and blood pressure detection. However, their narrow working pressure range and potential stability issues limit their use in dynamic or high‐pressure environments.

Micro‐roughness structures, by contrast, offer a broader working pressure range and enhanced stability under dynamic conditions. The roughened surface enhances contact reliability and pressure distribution, ensuring consistent performance under variable conditions. Although their sensitivity is moderate compared to nanoscale designs, micro‐roughness structures are more robust and durable under repetitive or abrasive conditions, making them suitable for robotic and environmental monitoring. However, maintaining the integrity of roughened surfaces during prolonged high‐frequency operations remains challenging, emphasizing the need for advanced materials.

Porous structures, renowned for their durability and adaptability, leverage interconnected networks to enhance mechanical flexibility and ion transport efficiency. These features ensure consistent performance over extended periods, making porous designs ideal for industrial sensing and environmental monitoring. Despite these strengths, challenges such as limited sensitivity and the difficulty of achieving scalable and uniform porosity remain significant. Addressing these limitations is critical for expanding their use in high‐precision applications.

Hierarchical structures integrate nanoscale sensitivity and microscale robustness, balancing high sensitivity with an extended working pressure range. These versatile designs enable multifunctional sensing, with particular applications in human–machine interaction and intelligent healthcare platforms. However, their fabrication complexity and the need to control interfacial properties across multiple scales pose significant challenges. Additionally, trade‐offs between response time and long‐term stability may occur, depending on material and structural choices.

The selection of sensor type should be tailored to meet specific application demands. For applications requiring high precision, such as healthcare monitoring, nanoscale structures are particularly advantageous due to their sensitivity to subtle pressure changes. In mechanically dynamic or high‐pressure environments, such as robotics and industrial sensing, micro‐roughness and porous structures offer enhanced durability and adaptability. Hierarchical structures, with their balanced sensitivity, durability, and multifunctionality, offer remarkable versatility for platforms like intelligent sensing systems.

When comparing these designs, the inherent trade‐offs among sensitivity, working range, and stability become evident, as these factors often interplay during performance optimization. Nanoscale structures achieve high sensitivity but are limited in their adaptability. Micro‐roughness and porous designs offer enhanced stability and robustness, while hierarchical structures are designed to balance these competing factors. Future research should explore hybrid designs that leverage the complementary strengths of different structures, such as nanoscale sensitivity with porous stability, to address diverse application requirements. Such advancements could pave the way for novel applications of EDL pressure sensors in complex and dynamic environments.

## 7 Applications of EDL Pressure Sensors

Smart structural designs and microengineering have facilitated the creation of EDL pressure sensors with high sensitivity, a broad linear range, stable performance, and rapid response capabilities. These advanced sensors meet emerging demands in diverse fields, including wearable healthcare electronics, environment and aerodynamic sensing, human–machine interaction, robotics, and ML‑enabled intelligent sensing platforms. The following sections highlight EDL pressure sensor applications in various fields.

### 7.1 Healthcare

Flexible EDL pressure sensors provide significant benefits for healthcare applications, particularly for monitoring physiological pressure variables. These sensors exhibit high sensitivity and real‐time response, enabling precise detection of subtle pressure variations and continuous data acquisition. This ensures accurate dynamic monitoring of physiological parameters. Additionally, their flexibility and high spatial resolution make them well‐suited for wearable and portable applications, offering comfort and precision to meet diverse clinical requirements.

**Figure 18:**
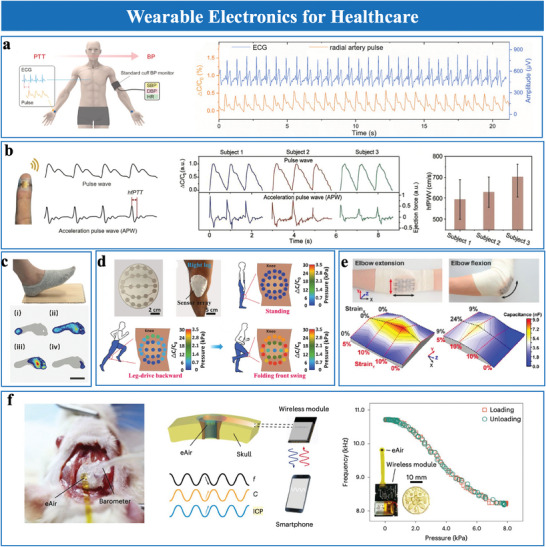
Applications of EDL pressure sensors in healthcare. a) Integration of ECG electrodes and iontronic sensor enables simultaneous monitoring of ECG signals and arterial pulses. Reproduced with permission.^[^ ^47^ ^]^ Copyright 2022, Elsevier. b) Fingertip pulse and acceleration pulse waves for subjects with varying degrees of arteriosclerosis, with hfPWV comparision. Reproduced with permission.^[^ ^71c^ ^]^ Copyright 2023, Wiley‐VCH. c) Plantar pressure distribution during a single stride. Reproduced with permission.^[^ ^22b^ ^]^ Copyright 2024, Wiley‐VCH. d) Wearable 5 ×5 sensor array mapping knee pressure distribution. Reproduced with permission.^[^ ^78c^ ^]^ Copyright 2022, Elsevier. e) Capacitance distribution mapping of elbow joint movement. Reproduced with permission.^[^ ^72a^ ^]^ Copyright 2021, American Chemical Society. f) Wireless intracranial pressure sensing on a rat cranium, including working principle and frequency‐pressure correlation. Reproduced with permission.^[^ ^95^ ^]^ Copyright 2023, Springer Nature.

A critical application is real‐time pulse wave monitoring, which is key to preventing arteriosclerosis‐related cardiovascular diseases.^[^ ^1a^ ^]^ For instance, arterial applanation tonometry via skin‐mounted sensor patches enables non‐invasive blood pressure measurement and arterial stiffness assessment through parameters like the augmentation index and pulse transit time (PTT).^[^ ^71c^ ^]^ Recent innovations integrate flexible iontronic sensors with Electrocardiogram (ECG) signals enabling continuous, real‐time blood pressure estimation via PTT, which tracks the time delay between the heart's electrical activity and the pulse wave reaching peripheral sites (**Figure** **18**a).^[^ ^47^ ^]^ This non‐invasive approach outperforms traditional cuff‐based blood pressure methods. Arteriosclerosis is also clinically assessed using pulse wave velocity (PWV), often necessitating bulky equipment.^[^ ^127^ ^]^ A new single‐point measurement strategy using flexible iontronic sensors supports fingertip pulse and heart‐fingertip PWV (hfPWV) measurement.^[^ ^71c^ ^]^ As illustrated in Figure 18b, sensor data enables the capture of fingertip pulse waveforms and accelerated pulse waves (APW), revealing the severity of arteriosclerosis through changes in T‐wave shifts and hfPWV increases. This approach offers an accessible, real‐time solution for arteriosclerosis diagnosis and monitoring. Besides blood pressure, these sensors are extensively used to monitor respiratory and heartbeat signals, aiding in the diagnosis of sudden death syndrome,^[^ ^53a^ ^]^ and identifying vibratory patterns associated with laryngeal disorders.^[^ ^70b^ ^]^ Their high resolution enables precise, localized monitoring, essential for accurate physiological assessments.

In addition to individual sensors, deploying sensor arrays plays a vital role in achieving high‐resolution spatial pressure mapping.^[^ ^13b^ ^]^ For example, Figure 18c shows plantar pressure distribution during walking, where the sensor array captures localized pressure variations across the foot, essential for gait analysis and abnormality detection.^[^ ^22b^ ^]^ Similarly, Figure 18d depicts dynamic pressure mapping on the knee during leg movements, such as the “leg‐drive backward” and “folding front swing”, showcasing its real‐time data capabilities throughout the gait cycle. Figure 18e highlights the sensor array's capability to track complex 3D strain distribution in the elbow during extension and flexion, essential for wearable systems in dynamic joint motion analysis.^[^ ^72a^ ^]^

Advancements in wireless communication have propelled system‐level wearable innovations, offering efficient approaches for personalized healthcare and remote monitoring. For instance, the bioinspired aero‐elastic capacitive pressure sensor (eAir) facilitates continuous real‐time intracranial pressure (ICP) monitoring, wirelessly transmitting data to a smartphone interface for remote observation (Figure 18f).^[^ ^95^ ^]^ This system offers a minimally invasive approach for prolonged ICP monitoring, enabling timely interventions for traumatic brain injury and hydrocephalus.

Beyond monitoring physiological variables, EDL pressure sensors are gaining prominence in clinical endoscopic imaging and surgeries. A transparent iontronic sensing‐enabled endoscope combines optical imaging with tactile sensing to quantitatively evaluate the stiffness contrast between normal and malignant tissues, presenting a novel multifunctional diagnostic solution.^[^ ^39a^ ^]^ Additionally, integrating sensors like eAir into laparoscopic tools enables real‐time tactile feedback during minimally invasive surgeries. This integration improves tissue handling precision, reduces damage, and significantly enhances surgical outcomes.^[^ ^95^ ^]^

In healthcare applications, EDL pressure sensors must meet task‐specific performance requirements. For blood pressure and pulse wave monitoring, high sensitivity and low detection limits are essential for capturing subtle physiological signals. Fast response and recovery times ensure accurate real‐time tracking of dynamic parameters, such as pulse transit time and gait patterns. Long‐term stability is crucial for continuous monitoring, like ICP measurement, while high spatial resolution supports precise pressure mapping in gait analysis and joint movement tracking. Furthermore, transparency and flexibility improve patient comfort and device usability in wearable devices. These key characteristics collectively enhance the efficacy of EDL sensors in personalized medicine, remote monitoring, and clinical diagnostics.

### 7.2 Environment and Aerodynamic Sensing

Flexible EDL pressure sensors provide critical benefits for environmental and aerodynamic sensing, including high sensitivity, real‐time response, and durability in dynamic environments. Accurately measuring subtle pressure variations in challenging conditions, such as fluctuating wind pressure or underwater environments, makes them ideal for continuous monitoring in natural and industrial applications.

**Figure 19:**
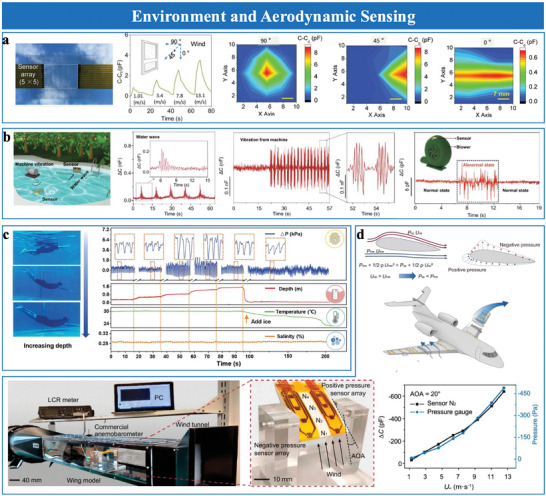
Applications of EDL pressure sensors for environment and aerodynamic sensing. a) 5 × 5 pixel sensor array integrated into a smart window for wind speed and direction detection, with mapped signals for 90°, 45°, and 0° wind directions. Reproduced with permission.^[^ ^118^ ^]^ Copyright 2020, Wiley‐VCH. b) Detection of water waves, underwater machine vibrations, and abnormal vibrations of blowers. Reproduced with permission.^[^ ^14^ ^]^ Copyright 2021, Springer Nature. c) Activities involving underwater suspension and diving across varying depths, paired with data recorded by the aquatic skin. Reproduced with permission.^[^ ^93^ ^]^ Copyright 2022, Wiley‐VCH. d) Sensor designed for measuring wind pressure on aircraft, showing pressure distribution across the wing surface, experiment setup, acquisition circuit, and comparison with a commercial pressure gauge. Reproduced with permission.^[^ ^31^ ^]^ Copyright 2024, Springer Nature.

A notable example is a transparent 5×5‐pixel sensor array mounted on a glass surface for wind intensity and direction detection. The sensor achieved precise measurement of wind velocities ranging 1.01–13.1 m s^−1^ and mapped directions at 0°, 45°, and 90° (**Figure** **19**a),^[^ ^118^ ^]^ highlighting its high spatial resolution and real‐time capabilities. These sensors are well‐suited for smart window applications, offering continuous wind monitoring and facilitating adaptive environmental controls.

The exceptional sensitivity of EDL sensors is critical in detecting subtle mechanical signals. For instance, Liu et al.^[^ ^14^ ^]^ applied these sensors in aquatic environments to monitor wave signals at the water–air interface (Figure 19b). The sensor detected repetitive wave patterns and vibration signals generated by underwater motors, showing exceptional sensitivity and temporal resolution, crucial for enabling real‐time environmental monitoring. Additionally, when mounted on a blower surface, the sensor identified abnormal noise patterns, facilitating early fault detection crucial for machinery maintenance and diagnostics.

Real‐time monitoring in aquatic environments represents a critical application. A multi‐parameter aquatic skin patch attached to a diver's instep allowed continuous monitoring of pulse, temperature, water depth, and salinity (Figure 19c).^[^ ^93^ ^]^ The sensor's versatile functionality allowed simultaneous capture of physiological and environmental data. This offered a comprehensive assessment that improved diver safety and environmental awareness. This capability emphasizes the importance of integrating multi‐sensing and real‐time data in complex scenarios.

For aerodynamic sensing, flexible iontronic sensors are employed for precise wind pressure measurements in wind tunnel experiments.^[^ ^94^ ^]^ Wang et al.^[^ ^31^ ^]^ developed a high‐resolution iontronic skin capturing pressure ranges from −100 to 600 kPa, achieving a resolution of 100 Pa (positive pressure) and −20 Pa (negative pressure) (Figure 19d). When laminated onto curved surfaces, such as the NACA‐0012 wing, it accurately measured pressure changes at various angles of attack and free stream velocities. The measurements demonstrated close agreement with commercial gauge data. This demonstrated the sensor's high accuracy and real‐time pressure monitoring capabilities, providing critical insights for aerodynamic design and aircraft optimization.

For environmental and aerodynamic sensing, EDL pressure sensors must satisfy a range of performance requirements customized for specific applications. High sensitivity is crucial for detecting subtle pressure variations in wind or water flows. A broad pressure range ensures compatibility with both low‐pressure wave detection and high‐pressure aerodynamic assessments. Real‐time response and high resolution are critical for precise, continuous monitoring in dynamic environments. Additionally, long‐term stability and robustness are crucial for reliable operation in harsh conditions, including underwater environments or high‐speed airflow scenarios. These capabilities allow EDL sensors to deliver precise and real‐time data for both environmental monitoring and industrial applications.

### 7.3 Human–Machine Interaction

**Figure 20:**
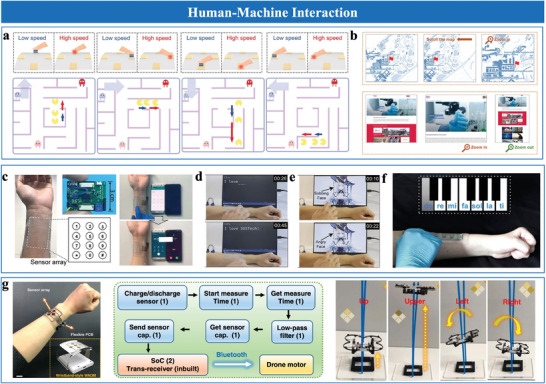
Applications of EDL pressure sensors for human–machine interaction. a,b) Dual mode HMIs equipped with sensor arrays support tasks such as control of virtual game characters, navigation of digital maps, and scrolling of documents. Reproduced with permission.^[^ ^21a^ ^]^ Copyright 2023, Wiley‐VCH. c) Transparent flexible pressure sensor arrays for making phone calls. Reproduced with permission.^[^ ^118^ ^]^ Copyright 2020, Wiley‐VCH. d,e) Transparent pressure sensors for typing and gaming. Reproduced with permission.^[^ ^50^ ^]^ Copyright 2023, Wiley‐VCH. f) Music control using pressure‐sensitive arrays. Reproduced with permission.^[^ ^128^ ^]^ Copyright 2021, Wiley‐VCH. g) Wearable aerial drone microcontroller. Reproduced with permission.^[^ ^24^ ^]^ Copyright 2019, Springer Nature.

Flexible EDL pressure sensors are well‐suited for human–machine interface applications due to their high sensitivity, wide pressure range, and adaptability. For example, a dual‐mode iontronic artificial skin array enables both tactile and touchless control modes. This design allows users to manipulate virtual game characters, navigate electronic maps, and scroll through documents, offering seamless and intuitive interaction to enhance user experience (**Figure** **20**a,b).^[^ ^21a^ ^]^ This dual‐mode functionality highlights the unique capability of iontronic pressure sensors to enable diverse control mechanisms, improving user engagement.

In addition to tactile control, flexible and transparent pressure sensor arrays have been integrated into wearable displays, such as a transparent keyboard for mobile devices, which allows users to make phone calls (Figure 20c),^[^ ^118^ ^]^ and interact with apps for typing,^[^ ^50^ ^]^ gaming,^[^ ^50^ ^]^ and music control.^[^ ^128^ ^]^ (Figure 20d–f) These applications underscore the versatility of iontronic sensors in portable and wearable electronics. Their integration into such devices is enabled by their high spatial resolution, allowing precise touch detection under static and dynamic conditions.

Moreover, wearable iontronic sensors, integrated with wireless and microcontroller modules, enable intuitive control of robotic systems.^[^ ^24^, ^50^, ^128^, ^129^ ^]^ By translating human gestures into electrical signals, these sensors enable seamless robot operation. For instance, a highly sensitive ionic mechanoreceptor skin, integrated with a wearable drone microcontroller, enables real‐time precision in direction and speed control (Figure 20g).^[^ ^24^ ^]^ This highlights their suitability for multi‐dimensional, dynamic applications.

For optimal performance in HMI applications, flexible EDL pressure sensors must satisfy critical requirements. High sensitivity is crucial for detecting subtle user inputs in virtual and touchless interactions, while rapid response and recovery times ensure seamless, real‐time control. Stability is essential for maintaining consistent performance over extended use, particularly in dynamic applications like robotic control that require continuous, multi‐dimensional input. Flexibility and transparency are vital for integration into wearable and portable devices, enabling unobtrusive interaction and precise touch detection. These characteristics highlight the potential of EDL sensors as a versatile platform for advancing HMI technologies across virtual controls and wearable robotics.

### 7.4 Robotic Interfaces

**Figure 21:**
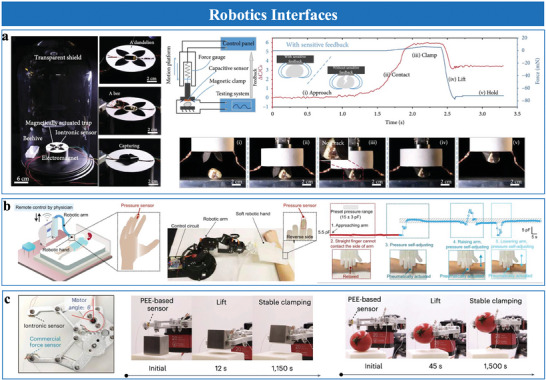
Applications of EDL pressure sensors in robotics interface. a) A smart autonomous system utilizing sensors as interfaces for sensory input and feedback. Reproduced with permission.^[^ ^27^ ^]^ Copyright 2022, Yongsong Luo et al. b) Stretchable pressure sensor for e‐skin and soft robotics. Reproduced with permission.^[^ ^29^ ^]^ Copyright 2021, The American Association for the Advancement of Science. c) Force sensing for precise and steady robotic manipulation. Reproduced with permission.^[^ ^88^ ^]^ Copyright 2024, Springer Nature.

The application of flexible EDL pressure sensors in robotics is advancing rapidly, significantly enhancing robots’ ability to sense their surroundings and perform complex tasks with greater precision. The flexibility, high sensitivity, and rapid response time of these sensors are particularly advantageous for human‐robot interaction, adaptive grasping, manipulation, and real‐time force control. For instance, Luo et al.^[^ ^27^ ^]^ developed an ultrasensitive iontronic sensor with a detection limit as low as 0.015 Pa, well‐suited for real‐time robotics environmental interactions. Integrated into a biomimetic Venus flytrap system, this sensor enabled the precise capture of small objects and safe handling of fragile items (**Figure** **21**a), facilitating intelligent interaction and enabling delicate manipulation. These highlight the distinctive capability of iontronic sensors to provide high precision in environments requiring fine control.

Additionally, Su et al.^[^ ^29^ ^]^ developed a stretchable, strain‐insensitive pressure sensor for real‐time tactile monitoring. When integrated into a robotic fingertip, the sensor exhibited stable performance under deformation of up to 50% strain. It could capture subtle pressure changes, such as arterial pulses, with high stability (Figure 21b). This capability makes the sensor well‐suited for applications in soft robotics, particularly in medical diagnostics and therapeutic devices. The sensor's ability to operate under significant strain without compromising accuracy or stability demonstrates the superior capability of iontronic sensors in managing dynamic and complex interaction scenarios.

Similarly, He et al. integrated an iontronic sensor into a robotic gripper, achieving precise force control during object manipulation.^[^ ^88^ ^]^ The sensor enabled stable grasping of a steel block at 350 kPa clamping pressure, while delicately manipulating fragile objects like cherry tomatoes (Figure 21c). This dual capability, stable grasping under high pressure, and sensitive manipulation at low pressures demonstrate the versatility of iontronic sensors in robotics, especially in applications requiring both strength and precision.

To ensure optimal performance in robotic applications, several key parameters are required. High sensitivity is crucial for detecting subtle pressure variations, enabling precise control in dynamic environments. Strain tolerance is essential to maintain sensor functionality under deformation, ensuring accurate feedback even during complex movements. Rapid response and recovery times are vital for providing real‐time tactile feedback, especially in tasks requiring quick adaptation. Long‐term stability ensures consistent performance over extended periods, particularly in delicate or repetitive tasks. Additionally, wireless communication capabilities enhance the adaptability of robotic systems by enabling seamless control and data transmission. High spatial resolution is also critical for precise force control and medical diagnostics.

### 7.5 ML‑Enabled Intelligent Sensing Platforms

Machine learning (ML) transforms sensing technology, optimizing data acquisition and interpretation.^[^ ^86^, ^130^ ^]^ When integrated with highly sensitive and flexible EDL pressure sensors, ML facilitates real‐time data processing and complex analysis. These capabilities are pivotal for applications in medical monitoring, robotics, and human–machine interfaces.

**Figure 22:**
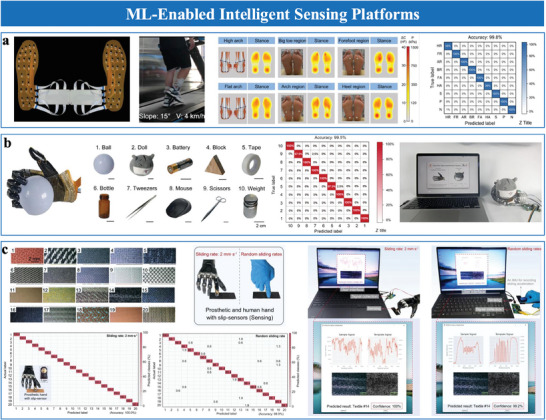
Applications of EDL pressure sensors for ML‑enabled intelligent sensing platforms. a) Flexible sensor system for plantar pressure distribution classification, with a confusion matrix showing 99.8% recognition accuracy across 9 tested objects. Reproduced with permission.^[^ ^103^ ^]^ Copyright 2024, American Chemical Society. b) Object recognition enabled by machine learning. Reproduced with permission.^[^ ^28^ ^]^ Copyright 2023, The American Association for the Advancement of Science. c) A sensory system for textile recognition, featuring portability and real‐time operation with a visual user interface. Reproduced with permission.^[^ ^20c^ ^]^ Copyright 2023, Springer Nature.

In healthcare, Li et al.^[^ ^103^ ^]^ developed a flexible iontronic pressure sensor integrated into a plantar pressure sensing system, achieving 99.8% accuracy in distinguishing foot types through deep learning algorithms. This system identifies foot arches and regions (e.g., big toe, arch, and heel) under different postures, making it effective for gait analysis and personalized healthcare interventions (**Figure** **22**a). This highlights the unique capability of EDL sensors to provide high spatial resolution and sensitivity, essential for dynamic and complex healthcare diagnostics. Similarly, Xu et al.^[^ ^108^ ^]^ employed in‐sensor dynamic deep learning integrated with a neuro‐inspired fully convolutional network (FCN) to assess knee motion during rehabilitation, demonstrating the potential of EDL sensors in clinical motion assessment and rehabilitation.

In robotics, the integration of sensor arrays with ML has advanced object recognition and manipulation tasks. For instance, a sensor array adhered to a prosthetic palm detected pressure distributions during objects grasping, achieving 99.5% accuracy in recognition using a 1D convolutional neural network (Figure 22b). This ML‐enhanced recognition system enables real‐time object classification, facilitating improved control and feedback in robotic manipulation. The ability to integrate high‐sensitivity EDL sensors with ML algorithms highlights the distinctive benefits of these sensors in dynamic and real‐time interactive applications.

Texture recognition represents another significant advancement. Bai et al.^[^ ^20c^ ^]^ developed an artificial sensory system using slip‐sensors and ML for texture classification, achieving 98.6% recognition accuracy for 20 textiles (Figure 22c). Recognizing subtle texture variations using highly sensitive EDL sensors enhances the performance of prosthetic devices and robotic systems, while advancing applications in virtual reality and consumer electronics.

To ensure the efficacy of these ML‐enabled platforms, several key performance parameters are necessary. High sensitivity is crucial for precise pressure detection and timely data capture, particularly in dynamic environments. Fast response and recovery times are crucial for enabling real‐time feedback and interaction, especially in robotic and healthcare applications. Additionally, advanced data processing capabilities are essential to manage complex ML tasks. Long‐term stability is critical to ensure consistent performance in continuous monitoring and high‐precision tasks. Finally, ML integration enables the adaptability required for real‐time and intelligent sensing across diverse applications, optimizing performance in response to evolving data streams.

## 8 Conclusion and Prospects

Over the past decade, flexible EDL pressure sensors have achieved remarkable progress, driven by advancements in materials and structural engineering. This review highlights recent innovations in morphological engineering of sensitive layers, focusing on representative microstructures. These include nano‐ and microscale designs, such as micro‐rough surfaces, internal porous structures, and multiscale hierarchical configurations. These engineered morphologies directly impact key performance metrics, including sensitivity, linear operating ranges, stability, response, hysteresis. Additionally, they enable unique features like transparency, wearability, directional selectivity, and multifunctionality. This review also explores fabrication strategies for tailored microstructures, including micropatterning and externally assisted techniques, as well as the emerging potential of fabricating stretchable and conformable EDL pressure sensors for complex 3D surfaces. Furthermore, a comprehensive comparison of sensor types is provided, offering guidelines for selecting suitable sensors. These advancements greatly enhance their adaptability in healthcare, environmental and aerodynamic monitoring, HMI, robotics, and intelligent sensing platforms.

Despite substantial progress, several critical challenges hinder the practical deployment of EDL pressure sensors. These include ensuring long‐term interface stability, achieving a balance between sensitivity and sensing range, decoupling multifunctional sensing, achieving strain‐insensitive performance, and advancing intelligent design and optimization. Addressing these issues requires a multidisciplinary effort, integrating advances in materials science, structural engineering, advanced manufacturing, and data‐driven design methodologies.

1. Enhancing Interface Stability

The interface stability of EDL pressure sensors is essential for consistent sensing performance, as it directly affects ionic transport, mechanical integrity, and long‐term reliability. Current strategies, such as material integration,^[^ ^102^ ^]^ microstructure optimization,^[^ ^28^ ^]^ and unified material systems,^[^ ^103^ ^]^ have improved mechanical durability and environmental stability. These approaches address issues like modulus mismatches, water loss, and phase transitions under extreme conditions.^[^ ^20^, ^58^, ^89^ ^]^ However, balancing these strategies to ensure long‐term ionic conductivity, structural flexibility, and environmental resilience remains a critical challenge. Emerging issues, such as ion migration under electric fields and interfacial degradation during prolonged operation, also require further exploration.^[^ ^131^ ^]^

Future advancements should focus on integrating adaptive materials, biomimetic designs, and advanced material systems to enhance interface stability. Stimuli‐responsive polymers and dynamic crosslinking networks offer self‐healing capabilities and adaptability to environmental fluctuations, ensuring long‐term reliability.^[^ ^89^, ^132^ ^]^ Biomimetic designs inspired by structures like octopus suckers can improve adhesion and evenly distribute stress, particularly under dynamic conditions.^[^ ^133^ ^]^ Advanced unified material systems, combining hierarchical architectures with intrinsic compatibility, could provide seamless interfaces that balance mechanical stability and environmental resilience. Additionally, thermally stable ionogels and advanced encapsulation techniques are essential for stable operation across a wide temperature range.^[^ ^122^, ^134^ ^]^ Controlling ion migration through electrostatically balanced networks or fixed ionic groups will address long‐term degradation. Aligning these strategies with application‐specific demands, such as biocompatibility for medical devices or extreme climate adaptability for environmental monitoring, will be critical for future progress.

2) Balancing High sensitivity and Wide Linear Range

High sensitivity enables EDL pressure sensors to accurately detect subtle pressure changes. High linearity reduces measurement errors, improves data reliability, and simplifies signal processing. A wide sensing range allows the sensor to adapt to varying pressure intensities, enabling use in diverse scenarios. Achieving these performance metrics simultaneously is a persistent challenge, requiring material and structural innovations to balance sensitivity, linearity, and range effectively.^[^ ^20^, ^70^ ^]^ Recent studies, such as gradient porous architectures, hierarchical multiscale designs, innovative material‐structure integrations have optimized deformation pathways to enhance low‐pressure sensitivity while maintaining linearity at higher pressures.^[^ ^17^, ^43^, ^79^, ^135^ ^]^ However, most of these strategies are still limited to specific ranges, reducing adaptability to dynamically varying pressures.^[^ ^18^, ^27^, ^79^ ^]^ Additionally, challenges in fabrication uniformity and scalability hinder their widespread application.

Future advancements should focus on adaptive microstructures and hybrid material systems. Adaptive microstructures, such as programmable gradient structures, can dynamically adjust deformation pathways to optimize sensitivity and range in real time.^[^ ^136^ ^]^ Hybrid material systems, such as dynamically crosslinked ionogels with nanoscale fillers, offer enhanced ionic transport and mechanical durability under varying pressures.^[^ ^137^ ^]^ Integrating these strategies with advanced manufacturing techniques, such as high‐resolution 3D printing, can overcome challenges in reproducibility and scalability, enabling precise control over complex structures like porous networks and hierarchical architectures.^[^ ^138^ ^]^

While balancing high sensitivity and wide sensing range is essential for broad applicability, many practical applications require targeted performance within specific pressure ranges. Low‐pressure scenarios, such as wearable healthcare monitoring, prioritize ultrahigh sensitivity for detecting subtle physiological signals. In contrast, high‐pressure environments, such as industrial monitoring and aerospace applications, demand consistent sensitivity and structural stability.^[^ ^31^ ^]^ Current designs often focus on low‐pressure optimization, leaving high‐pressure performance underdeveloped. Future research should address this gap by enhancing high‐pressure sensitivity through robust hierarchical architectures with stress‐distributing configurations. These designs can prevent signal saturation and maintain linear responses under extreme conditions. Such advancements will enable EDL sensors to meet diverse application demands, including precise intraocular and intracranial pressure monitoring, and real‐time stress detection in industrial and aerospace settings.

3) Multifunctional Sensing with Decoupling Capabilities

Multimodal sensing is essential for EDL sensors to address diverse application demands. However, integrating multiple sensing modalities (e.g., pressure and temperature) within a single platform faces challenges such as overlapping signal outputs, cross‐talk, and nonlinear interactions.^[^ ^91^, ^92^ ^]^ Current strategies, including vertical layering and horizontal compartmentalization, have partially addressed these issues by isolating sensing functions through spatial separation or distinct mechanisms.^[^ ^42^, ^93^ ^]^ Nevertheless, these approaches are often limited in scalability and adaptability under dynamic conditions, necessitating advancements in material innovations, structural optimizations, and signal processing techniques.

Future advancements should prioritize material innovations and structural designs tailored for multimodal decoupling.^[^ ^139^ ^]^ Dynamic hybrid ionic gels, integrating pressure‐sensitive and thermally responsive regions, enable selective amplification of distinct signal modalities. For example, dynamic cross‐linked ionic networks with tunable ionic conductivity can adapt to mechanical deformation for pressure detection, while responding to temperature variations through thermally induced ion mobility changes. Incorporating high‐permittivity materials into these ionic gels can further enhance thermal sensitivity without compromising pressure detection. This approach leverages material‐specific responses to create independent sensing pathways, enabling effective decoupling of pressure and temperature. Structural designs must move beyond traditional vertical layering and horizontal compartmentalization to address the complexities of multimodal sensing.^[^ ^140^ ^]^ Multiscale architectures, combining microstructured porous layers with nanoscale thermoresponsive films, provide an integrated solution for multimodal sensing. For instance, deformable microstructures can enhance interfacial capacitance, improving pressure sensitivity, while nanoscale functional layers selectively respond to thermal stimuli. Spatially separating these functional domains and optimizing their interface interactions minimize cross‐talk and improve sensing precision in multimodal systems. Moreover, flexible strain‐isolation layers can be incorporated to reduce mechanical interference between sensing regions, ensuring consistent performance under dynamic conditions.

Signal processing will play a critical role in real‐time signal decoupling.^[^ ^141^ ^]^ Techniques such as time‐domain filtering can isolate rapid pressure fluctuations from slower thermal responses, while frequency‐domain approaches leverage distinct spectral properties for different stimuli. For example, assigning pressure signals to low‐frequency bands and temperature signals to high‐frequency bands can enhance signal separation in dynamic conditions. Intelligent algorithms, such as machine learning models trained on multimodal datasets, further enable real‐time extraction of independent signal contributions, even under noisy conditions.^[^ ^139b^ ^]^ Hardware‐accelerated platforms integrated with machine learning will support scalable and robust signal processing for real‐world applications.

4) Achieving Strain‐Insensitive Performance

Mechanical deformations, such as stretching and bending, pose significant challenges to the performance of EDL pressure sensors, particularly when conforming to dynamic and irregular surfaces. These deformations can cause changes in the contact area between the electrode and dielectric layer, leading to capacitance fluctuations and signal interference. Addressing these challenges is essential to ensure reliable performance in dynamic and wearable applications.

Structural strategies offer effective solutions to mitigate strain‐induced performance fluctuations. Rigid microstructures, such as pyramid‐shaped elements, stabilize the contact area by maintaining the geometry of the sensing region and ensuring consistent contact between the electrode and dielectric layer, even under dynamic deformation.^[^ ^29^, ^142^ ^]^ This design isolates strain effects and enables stable electrical performance. Building on this, hierarchical or multi‐layered architectures further enhance stability by distributing mechanical stress across rigid and flexible layers.^[^ ^143^ ^]^ While rigid layers absorb most of the stress, flexible layers accommodate deformation, protecting strain‐sensitive regions and maintaining reliable performance in dynamic strain environments.

In addition to structural design strategies, material innovations offer significant potential for mitigating strain effects in EDL pressure sensors. Elastic dielectrics with enhanced mechanical properties, such as nanofiber‐reinforced PDMS or graphene‐based composites,^[^ ^144^ ^]^ effectively decouple mechanical strain from sensing performance by maintaining dielectric stability and preventing localized deformation. These materials ensure consistent capacitance and precise pressure sensing even under significant strain. The incorporation of advanced 2D materials, such as MXenes, further strengthens the dielectric layer by improving both its mechanical resilience and electrical conductivity, thereby enhancing the sensor's durability and overall performance.

Dynamic buffering mechanisms, such as flexible spacers, complement these material strategies by maintaining a consistent dielectric gap during deformation.^[^ ^145^ ^]^ By absorbing strain‐induced stresses, these spacers stabilize the sensor's electrical output and improve its reliability and precision in dynamic and wearable applications. Together, these material and buffering strategies provide a robust foundation for the development of strain‐insensitive EDL pressure sensors.

5) Intelligent design and optimization

The design of EDL pressure sensors involves balancing trade‐offs between performance parameters, such as sensitivity, linearity, and durability, challenges traditionally addressed through time‐consuming trial‐and‐error methods. Recent advancements in data‐driven methodologies, such as inverse design and application‐guided optimization, offer a more efficient alternative by directly predicting optimal microstructures and material parameters based on desired performance outputs.^[^ ^86d^ ^]^ These approaches, leveraging machine learning models, reduced‐order modeling, and jumping‐selection techniques, significantly accelerate the design process while minimizing data requirements.

In the context of EDL sensors, intelligent design methods can address unique challenges, such as optimizing multiscale microstructures to balance sensitivity and linearity or ensuring long‐term stability under dynamic environmental conditions. For example, machine learning algorithms can predict and refine gradient porous architectures to enhance deformation control, while jumping‐selection techniques enable the identification of robust configurations for improved scalability and reproducibility. By integrating these tools with advanced manufacturing techniques, such as high‐resolution 3D printing, researchers can efficiently prototype scalable and high‐performance EDL sensors tailored for specific applications. Future advancements should focus on expanding the scope of these intelligent methods, such as incorporating multimodal datasets to address complex requirements like multi‐functionality or extreme environmental resilience. These strategies will enable the development of EDL sensors with unprecedented adaptability and precision, bridging the gap between laboratory research and real‐world deployment in fields such as healthcare, robotics, and aerospace.

## Conflict of Interest

The authors declare no conflict of interest.
